# Microglia-derived nanovesicles synchronize macroautophagy and chaperone-mediated autophagy for Alzheimer’s disease therapy

**DOI:** 10.1038/s41392-025-02453-y

**Published:** 2025-11-03

**Authors:** Min Li, Shuang Chen, Rong Guo, Yang Wang, Mingrui Yang, Yingke Liu, Qiang Zhang, Shiyu Zhu, Jiaxin Li, Fang Chen, Bo Wang, Man Li, Qin He

**Affiliations:** 1 Key Laboratory of Drug-Targeting and Drug Delivery System of the Education Ministry and Sichuan Province, Sichuan Engineering Laboratory for Plant-Sourced Drug and Sichuan Research Center for Drug Precision Industrial Technology, West China School of Pharmacy, Sichuan University, Chengdu, PR China; 2 Department of Biochemistry and Molecular Biology, West China School of Basic Medical Sciences and Forensic Medicine, Sichuan University, Chengdu, PR China; 3 Department of Medical Biochemistry and Biophysics, Karolinska Institute, Stockholm, Sweden; 4 State Key Laboratory of Oral Diseases, National Clinical Research Center for Oral Diseases, West China Hospital of Stomatology, Sichuan University, Chengdu, PR China

**Keywords:** Diseases of the nervous system, Blood-brain barrier

## Abstract

Dysregulated autophagy is a hallmark of Alzheimer’s disease (AD), yet the extent of impairment in macroautophagy and chaperone-mediated autophagy (CMA) remains unclear. Here, we show that both pathways are disrupted in AD model mice, preceding β-amyloid accumulation and driving disease progression. However, therapeutic autophagy modulation is severely restricted by the blood–brain barrier (BBB). To overcome this, we developed Microglia-Liposome Fusion Extrusion (MiLi-FE), a method to engineer microglia-derived nanovesicles (AR@ENV) for the codelivery of AR7 (a CMA inducer) and rapamycin (a macroautophagy inducer). Leveraging its microglial membrane origin, AR@ENV effectively crosses the BBB and targets inflammatory sites in the AD brain, where it is internalized by neurons. Once inside, they synchronously activate both autophagy pathways: AR7 antagonizes retinoic acid receptor alpha (RARα) to enhance CMA, while rapamycin inhibits mTOR to promote macroautophagy. This coordinated activation enhances clearance of β-amyloid and other toxic aggregates, restores proteostasis, and provides robust neuroprotection. Furthermore, the strategy ameliorates neuroinflammation and significantly rescues cognitive deficits in two distinct AD mouse models. By integrating synchronized dual autophagy activation with targeted biomimetic delivery, AR@ENV represents a promising therapeutic candidate for AD. Moreover, the MiLi-FE platform offers a versatile and scalable approach for delivering diverse therapeutics to the central nervous system, extending its potential applicability to a range of neurological disorders.

## Introduction

Alzheimer’s disease (AD) is one of the most prevalent neurodegenerative disorders and is characterized by two pathological hallmarks: the extracellular deposition of β-amyloid (Aβ) plaques, which aggregate with various proteins to form senile plaques, and the intracellular accumulation of hyperphosphorylated tau protein, leading to neurofibrillary tangles.^1–3^ These alterations impair neuronal function, promote toxic protein aggregation, disrupt metal ion homeostasis, and trigger inflammatory responses, all of which contribute to disease progression.^4–6^ Currently approved pharmacological treatments for AD, such as acetylcholinesterase inhibitors (e.g., Donepezil) and NMDA receptor antagonists (e.g., Memantine), provide only symptomatic relief without altering disease progression.^7^ In contrast, the emergence of anti-amyloid monoclonal antibodies (MAbs), including Aducanumab, Lecanemab, Donanemab, and Gantenerumab, marks a shift toward disease-modifying strategies.^8^ These MAbs reduce amyloid-β (Aβ) plaque burden and slow cognitive decline in some patients; however, they are associated with significant limitations: Low blood-brain barrier (BBB) penetration, necessitating high systemic does^9,10^; Lack of effect on de novo Aβ generation and limited impact on tau pathology.^8,11^ Similarly, emerging anti-tau therapies, including kinase inhibitors and tau aggregation blockers, face challenges related to off-target toxicity and poor clinical efficacy.^12^

A central factor in Alzheimer’s disease (AD) pathology is the impairment of neuronal proteostasis, a process critically regulated by autophagy—an essential intracellular degradation pathway.^5,13^ Autophagy plays a key role in clearing toxic protein aggregates, such as Aβ and tau, and its dysregulation has been strongly implicated in AD pathogenesis.^5,13^ Growing evidence suggests that autophagy dysfunction may occur even prior to the deposition of Aβ plaques, highlighting its potential role in the early stages of the disease.^14^ While the majority of previous studies have focused on macroautophagy, a non-selective bulk degradation process, recent findings underscore the important complementary role of chaperone-mediated autophagy (CMA).^15–18^ CMA is a highly selective lysosomal degradation pathway that recognizes and degrades specific pathogenic proteins containing KFERQ-like motifs, including certain forms of tau and amyloid precursor protein (APP).^15–18^ Impairment of CMA contributes to proteomic instability and accumulation of abnormal proteins, thereby accelerating neurodegenerative processes.^15–18^ Given the coordinated roles of macroautophagy and CMA in maintaining cellular homeostasis, simultaneous activation of both pathways has emerged as a promising therapeutic strategy for AD. is,^19^ Enhancing both forms of autophagy may increase overall autophagic flux, promoting efficient clearance of multiple pathogenic proteins and potentially alleviating neurotoxicity caused by protein misfolding and aggregation. This combined approach holds promise for slowing or even reversing disease progression.

**Fig. 1:**
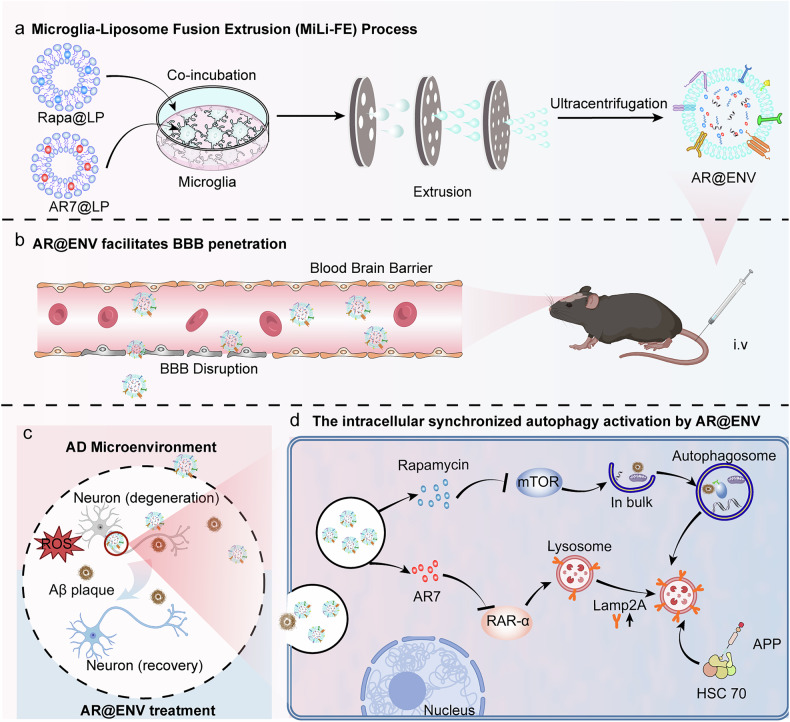
Schematic representation of AR@ENV fabrication via the Microglia-Liposome Fusion Extrusion (MiLi-FE) process and its role in autophagy regulation in the AD Brain. **a** Illustration of the MiLi-FE process for AR@ENV generation. Prepared Rapa@LP (Rapamycin-loaded liposomes) and AR7@LP (AR7-loaded liposomes) were incubated with microglia (BV2 cells). After internalization of the liposomes by microglia, the drug-loaded microglia are then extruded through nanoporous membranes, yielding AR@ENV. **b** Illustration of the ability of AR@ENV to cross the compromised BBB associated with AD pathology after intravenous (i.v.) injection. **c** AR@ENV targets inflammatory sites and facilitates neuronal repair within the pathological microenvironment of AD brains. **d** During internalization by neuronal cells, AR@ENV releases rapamycin and AR7, synchronizing the activation of macroautophagy and CMA. Rapamycin induces macroautophagy via mTOR inhibition, facilitating the clearance of neurotoxic proteins and damaged cellular components. AR7 enhances CMA by inhibiting RAR-α, restoring the degradation of precursor proteins linked to AD-associated toxic aggregates and thereby reducing protein misfolding and cytotoxicity. Created with BioRender

Despite the therapeutic potential of autophagy modulation, a major challenge is the efficient delivery of interventions to the brain, as the blood‒brain barrier (BBB) severely restricts drug uptake into the central nervous system (CNS), limiting the effectiveness of many promising treatments.^20–22^ Microglia, the resident immune cells of the brain, can infiltrate the BBB and migrate toward pathological sites during neuroinflammation,^23,24^ making their membrane-derived vesicles promising candidates for neuron-targeted drug delivery.^25,26^ However, the direct use of microglia-derived vesicles face challenges, including low production yield, high isolation costs, and batch-to-batch variability, hindering large-scale therapeutic applications.^27,28^ To address these challenges, we developed Microglia-Liposome Fusion Extrusion (MiLi-FE), a bioengineering strategy that integrates liposome-based drug encapsulation with microglia-derived nanovesicle production. Using this approach, we engineered AR@ENV, a microglia-derived nanovesicle coloaded with AR7 and rapamycin, for autophagy modulation in AD therapy (Fig. 1a, b). In this process, AR7 and rapamycin—both highly hydrophobic compounds—are first encapsulated in separate liposomes, which increase their solubility, improve their cellular uptake, and enable their controlled release. Liposome formulation facilitates microglial internalization during coincubation, leveraging their innate endocytic capacity, which would otherwise be inefficient for free hydrophobic drugs. The drug-loaded microglia are then extruded through nanoporous membranes to yield biomimetic nanovesicles enriched with microglial membrane proteins, retaining their ability to cross the BBB. This process enhances vesicle yield while preserving targeting specificity, biocompatibility, and dual-drug delivery capability, offering a scalable platform for synchronized CMA and macroautophagy activation in AD therapy.

Designed with these multifaceted capabilities, AR@ENV represents a targeted therapeutic nanoplatform engineered to overcome critical biological barriers in the treatment of Alzheimer’s disease. Its structural and functional properties are optimized to facilitate efficient traversal across the blood-brain barrier (BBB), achieve site-specific delivery to regions rich in amyloid-beta plaques and neurofibrillary tangles, and promote enhanced uptake by neurons and other affected neural cells (Fig. 1c). Upon reaching the brain parenchyma, AR@ENV undergoes controlled release of its dual autophagy-modulating payload: AR7 and rapamycin. This combination strategy is designed to concurrently address proteostatic dysfunction through complementary mechanisms. AR7, a potent and selective antagonist of retinoic acid receptor alpha (RAR-α), activates chaperone-mediated autophagy (CMA), enhancing the targeted degradation of pathogenic species such as phosphorylated tau and amyloid precursor protein derivatives. Simultaneously, rapamycin, a classical mTOR inhibitor, induces macroautophagy, bolstering the bulk clearance of insoluble protein aggregates and dysfunctional organelles (Fig. 1d). This synchronized activation of both CMA and macroautophagy is anticipated to improve proteostasis and cognitive function in models of Alzheimer’s disease (AD).

## Results

### Autophagy dysfunction in an AD mouse model

**Fig. 2:**
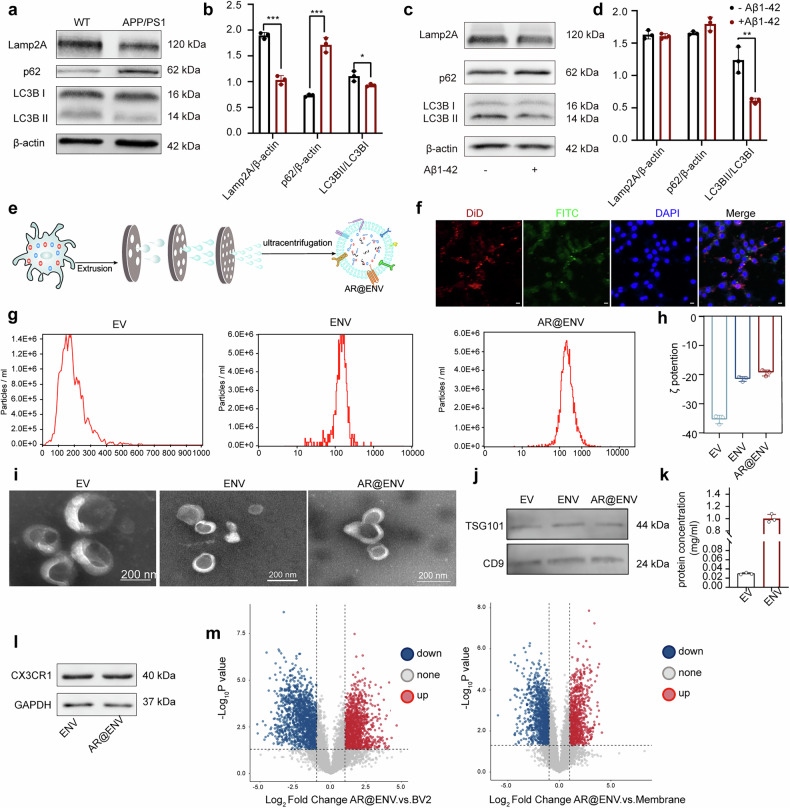
Generation and characterization of AR@ENV. **a** Western blot analysis of CMA- and macroautophagy-specific proteins (Lamp2A, p62, and LC3B) in the brains of APP/PS1 transgenic mice. **b** Semiquantitative analysis of protein bands from (**a**). **c** Western blot analysis of Lamp2A, p62, and LC3B expression in HT22 cells treated with Aβ1-42 oligomers. **d** Semiquantitative analysis of protein bands from (**c**). **e** Schematic representation of the MiLi-FE process for AR@ENV generation. **f** Intracellular status of dyes delivered by liposomes in microglia (BV2 cells). Scale bar = 20 μm. **g** Size distribution analysis. **h** ζ-Potential measurements. **i** TEM images. **j** Western blot analysis of vesicle-specific protein expression. **k** Comparative yields of EVs and ENVs. For all the bar plots, the data are presented as the means ± SDs, *n* = 3. **l** Western blot analysis of the BV2-specific protein CX3CR1 in ENVs and AR@ENVs. **m** Volcano plots of differentially expressed proteins across comparison groups. ENV vs. whole BV2 cells (Left). ENV vs. membrane (right). Red and blue dots denote significantly upregulated and downregulated proteins, respectively, and the data are presented as the means ± SDs, *n* = 3

The APP/PS1 mouse model is a well-established model for AD.^29^ To evaluate autophagy dysfunction in AD, we assessed the expression of macroautophagy- and CMA-associated proteins in APP/PS1 mouse brains. Compared with that in wild-type (WT) mice, the CMA-related protein Lamp2A was markedly reduced (Fig. 2a, b). Concurrently, the LC3BII/LC3BI ratio, an indicator of macroautophagy activity, was significantly decreased, whereas p62 levels were elevated, suggesting impaired autophagic flux. These findings indicate that both macroautophagy and CMA are compromised in AD.

To further investigate whether toxic proteins contribute to autophagy dysfunction, we established an Aβ1‒42 oligomer-induced HT22 cell model in vitro. Aβ1‒42 treatment reduced the LC3BII/LC3BI ratio but had no effect on p62 or Lamp2A expression levels (Fig. 2c, d). These results suggest that CMA and macroautophagy impairment may not be solely caused by toxic Aβ proteins. Instead, as previous studies have suggested,^14^ autophagy dysfunction may precede Aβ deposition, whereas Aβ1‒42 oligomers may accelerate macroautophagy impairment.

### MiLi-FE-based fabrication and characterization of AR@ENV

Given the observed dual impairment of macroautophagy and CMA in AD, therapeutic strategies that restore both pathways may help counteract disease progression. However, a major challenge in autophagy-targeting therapies is efficient drug delivery across the BBB, which severely limits the bioavailability of many promising modulators. To overcome this, we sought to develop a biomimetic nanodrug delivery system capable of synchronizing CMA and macroautophagy activation while efficiently crossing the BBB. To achieve this goal, we developed Microglia-Liposome Fusion Extrusion (MiLi-FE), a bioengineering strategy for generating microglia-derived nanovesicles that encapsulate and codeliver therapeutic agents.

In the MiLi-FE process, AR7 and rapamycin, both highly hydrophobic, were separately encapsulated into liposomal formulations (AR7@LP and Rapa@LP) to increase their solubility and delivery efficiency. Microglia were incubated with these drug-loaded liposomes, facilitating internalization, followed by extrusion through nanoporous membranes to generate AR@ENV nanovesicles that retain microglial membrane properties while incorporating encapsulated drugs (Fig. 2e). miLi-FE improves drug loading efficiency while preserving the innate targeting capabilities of microglia, enabling the precise delivery of autophagy modulators across the BBB.

Characterization of AR7@LP and Rapa@LP revealed particle sizes of 160.3 ± 3.4 nm and 127.3 ± 2.6 nm, respectively (Supplementary Fig. 1a, b), with polydispersity indices (PDIs) of ~0.2, indicating a uniform size distribution. The encapsulation efficiencies were 92.9 ± 1.3% (AR7@LP) and 84.5 ± 1.5% (Rapa@LP), while the drug loading efficiencies were 1.30 ± 0.012% and 3.91 ± 0.58%, respectively (Supplementary Table 1). Zeta potential analysis revealed negative surface charges of −37.9 ± 4.6 mV (AR7@LP) and −30.8 ± 1.5 mV (Rapa@LP) (Supplementary Fig. 1c, d). Transmission electron microscopy (TEM) imaging confirmed a well-defined phospholipid bilayer structure with a regular spherical morphology (Supplementary Fig. 1a, b).

The optimal drug loading time of 8 h was determined through comprehensive evaluation via a CCK8 assay, liposome‒lysosome colocalization analysis, and HPLC quantification of drug loading efficiency (Supplementary Figs. 2, 3 and Supplementary Table 2). To determine the distribution of encapsulated drugs within AR@ENV, we performed confocal microscopy using FITC- and DiD-labeled liposomes. The lack of significant colocalization of fluorescent markers in microglia indicated that AR7 and rapamycin were present in free form within AR@ENV rather than being retained within liposomes (Fig. 2f).

To compare AE@ENV with naturally secreted extracellular vesicles (EVs), we isolated EVs via ultracentrifugation. Nanoparticle tracking analysis (NTA) revealed that the diameters of the EVs, drug-free extruded nanovesicles (ENVs), and AR@ENVs were 175.4 ± 6.5 nm, 142.6 ± 5.2 nm, and 134.0 ± 4.7 nm, respectively (Fig. 2g). Zeta potential measurements revealed values of −35.33 ± 0.68 mV (EV), −21.42 ± 0.95 mV (ENV), and −19.40 ± 1.04 mV (AR@ENV) (Fig. 2h). TEM confirmed that ENVs and AR@ENVs maintained a disc-like morphology similar to that of EVs (Fig. 2i). Western blot analysis confirmed the presence of the EV-associated marker proteins TSG101 and CD9 (Fig. 2j), confirming the vesicular nature of AR@ENV.

Notably, the MiLi-FE process significantly increased the vesicle yield, with AR@ENV production being 33.8-fold greater than that of EVs (Fig. 2k). Furthermore, the drug-loading capacities of AR7 and rapamycin in AR@ENV were 19.9 ± 0.22 μg/mg and 7.02 ± 0.34 μg/mg, respectively (Supplementary Table 3), demonstrating high drug-loading efficiency via the MiLi-FE method.

The CX3CL1‒CX3CR1 axis represents a crucial signaling pathway between neurons and microglia and is essential for the neuronal targeting of AR@ENV. CX3CR1 expression in AR@ENV was examined via Western blotting. The results demonstrated that ENVs retain the CX3CR1 protein derived from microglia, with no effect on CX3CR1 expression observed during the drug loading process (Fig. 2l).

Furthermore, the complete proteome of AR@ENV was analyzed via proteomics. Proteomic analysis revealed a total of 7771 proteins (Supplementary Table 4). Specifically, 7206 proteins were detected in AR@ENV, 7058 in BV2 cells, and 6963 in microglial plasma membrane fractions (Supplementary Fig. 4). The number of proteins detected in AR@ENV cells was even greater than that detected in BV2 cells. This may be attributed to the retention of low-abundance proteins during the extrusion preparation of ENV, leading to an increase in their relative concentration and subsequent detection, whereas these proteins were undetectable in whole BV2 cells due to their low abundance.

Principal component analysis (PCA) further revealed noticeable shifts in the main components (PC1 and PC2) among samples derived from microglia, microglial plasma membranes, and AR@ENV (Supplementary Fig. 5).

Subsequent comparative analysis revealed protein changes in AR@ENV relative to whole BV2 cells and microglial plasma membrane fractions. Compared with those in whole BV2 cells (6931 proteins analyzed), 1792 proteins were upregulated (including 1396 with a ≥2-fold increase), 2074 were downregulated (including 1625 with a ≥2-fold decrease), and 164 proteins expressed in BV2 cells were not detected in AR@ENVs (Fig. 2m and Supporting Information Supplementary Table 5). Compared with those in microglial plasma membrane fractions (6836 proteins analyzed), 1469 proteins were upregulated (including 1018 with a ≥2-fold increase), 1820 were downregulated (including 1187 with a ≥2-fold decrease), and 78 proteins expressed on the microglial plasma membrane were not detected in ENVs (Fig. 2m).

### AR@ENV increases autophagy to achieve neuroprotective effects

**Fig. 3:**
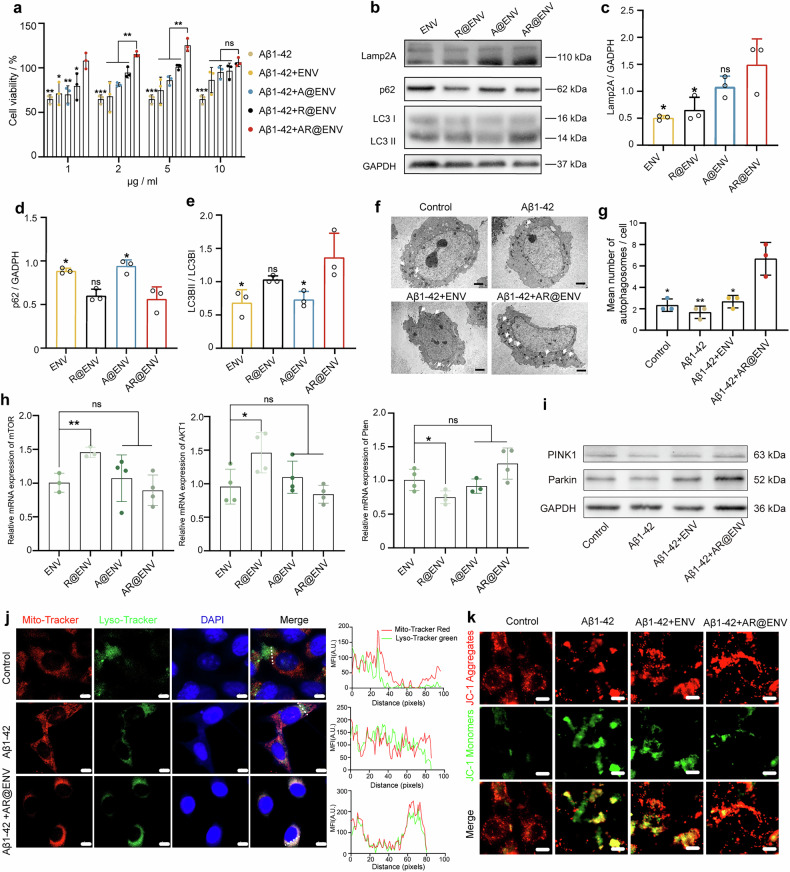
AR@ENV increases autophagy to exert neuroprotective effects. **a** Cytoprotective effect of AR@ENV against Aβ1‒42 oligomer-induced toxicity in HT22 neuronal cells. **b**–**e** Western blot analysis of CMA- and autophagy-related proteins (Lamp2A, p62, and LC3BII/LC3BI) in HT22 neuronal cells after treatment with ENV, A@ENV, R@ENV, or AR@ENV. **b** Representative Western blot images. **c** Semiquantitative analysis of Lamp2A (CMA marker). **d** Semiquantitative analysis of p62 (a macroautophagy marker). **e** Semiquantitative analysis of the LC3BII/LC3BI ratio. **f**, **g** TEM analysis of autophagosome formation in neuronal cells treated with different formulations. **f** Representative TEM images of the cytoplasm; the autophagosomes are indicated by arrows. Scale bar = 200 nm. **g** Quantification of autophagosomes per cell. **h** qPCR analysis of the effects of AR@ENV on core genes (mTOR, AKT1, and Pten) in the mTOR signaling pathway. The data are presented as the means ± SDs, *n* = 4. **i** Representative Western blot images showing the expression of the mitophagy-associated proteins PINK1 and Parkin in HT22 cells subjected to various treatments. **j** Colocalization of mitochondria (red) and lysosomes (green) in HT22 neuronal cells pretreated with or without AR@ENV, followed by Aβ1‒42 oligomer exposure. Nuclei were stained with DAPI (blue), and colocalization points are shown in yellow. Scale bar = 5 μm. A representative quantitative analysis of the mean fluorescence intensity (MFI) of the mitochondrial and lysosomal tracers is shown on the right. **K** Representative images of JC-1 aggregates (red) and monomers (green), indicating the mitochondrial membrane potential. Scale bar: 5 μm. For all the bar plots, the data are presented as the means ± SDs, *n* = 3

We next sought to evaluate whether AR@ENV could exert functional effects in a disease-relevant context. Since Aβ1–42 oligomers are known to induce neurotoxicity,^30^ we investigated whether AR@ENV could counteract Aβ1–42-mediated damage by restoring autophagy pathways and protecting neuronal cells. HT22 cells, a well-established neuronal cell model, were used to assess the effects of nanovesicles at various concentrations on Aβ1–42-induced neurotoxicity. After 24 h of Aβ1–42 exposure, the cell viability decreased to 59% (Fig. 3a). However, treatment with nanovesicles significantly improved cell survival, with AR@ENV demonstrating the most pronounced protective effect and even promoting cell proliferation (Fig. 3a). This enhancement may be attributed to the ability of AR@ENV to accelerate the clearance of toxic proteins, thereby facilitating normal cell cycle progression. Notably, higher concentrations of ENV also mitigated Aβ1‒42-induced cytotoxicity, potentially through the retention of microglial properties that enable effective Aβ1‒42 oligomer degradation.^31^

To determine whether AR@ENV upregulates autophagy, we performed western blot analysis to assess autophagy-related protein expression in HT22 neurons treated with different nanovesicles (Fig. 3b). Compared with the ENV and R@ENV groups, the A@ENV and AR@ENV groups presented a significant increase in Lamp2A expression (Fig. 3c), indicating CMA activation. Moreover, p62 levels decreased in the R@ENV and AR@ENV groups (Fig. 3d), whereas the LC3II/LC3I ratio, a hallmark of macroautophagy activation,^32^ was significantly elevated (Fig. 3e). These findings confirm that AR@ENV effectively activates macroautophagy in neuronal cells.

As demonstrated in our previous experiments, macroautophagy impairment is not solely caused by Aβ toxicity. TEM analysis revealed a significant reduction in the number of autophagosomes in Aβ1-42-treated neurons, indicating that autophagy was suppressed (Fig. 3f, g). ENV treatment did not increase autophagosome formation, suggesting a lack of autophagy-activating properties. In contrast, AR@ENV treatment significantly increased the number of autophagosomes, confirming its ability to increase macroautophagy in neuronal cells.

Given the established impact of rapamycin-induced macroautophagy on mTOR signaling, q‒PCR analysis was performed to evaluate the effects of AR@ENV on core mTOR pathway gene expression. Following 48 h of Aβ1‒42 induction, the results indicated a significant increase in the expression of mTOR and AKT1, alongside a decrease in Pten gene expression, which is indicative of mTOR pathway activation. However, the addition of AR@ENV suppressed this activation, potentially due to mTOR pathway inhibition resulting from autophagy activation. Moreover, ENV alone inhibited this activation, which was likely attributable to the high expression of autophagy-related proteins within ENVs (Fig. 3h).

In addition to its role in Aβ clearance, autophagy dysfunction in AD also contributes to mitochondrial damage, leading to reactive oxygen species (ROS) accumulation, neuronal apoptosis, and neuroinflammation.^19^ To assess whether AR@ENV affects mitophagy, its impact on Aβ1–42-treated HT22 neuronal cells was analyzed via combined Western blotting and mitochondrial–lysosomal colocalization assays. AR@ENV significantly upregulated the expression of the mitophagy-related proteins PINK1 and Parkin (Fig. 3i and Supplementary Fig. 6). Furthermore, confocal microscopy revealed increased mitochondrial–lysosomal colocalization signals upon AR@ENV treatment, collectively indicating enhanced mitophagy (Fig. 3j). Moreover, AR@ENV effectively protected against Aβ1‒42-induced mitochondrial impairment, leading to recovery of the mitochondrial membrane potential (Fig. 3k).

In summary, in vitro pharmacological assays demonstrated that AR@ENV activates both macroautophagy and CMA in neuronal cells. Additionally, AR@ENV protects neurons from Aβ1‒42-induced mitochondrial dysfunction by restoring mitochondrial autophagy, highlighting its therapeutic potential in AD.

### Microglia-derived ENVs facilitate BBB penetration and neuronal targeting

While AR@ENV has neuroprotective effects in vitro, its therapeutic success relies on efficient brain delivery, which is hindered by the restrictive nature of the BBB.^33,34^ We therefore investigated whether microglia-derived ENV, the precursor of AR@ENV, could effectively cross the BBB and reach neuronal cells.

**Fig. 4:**
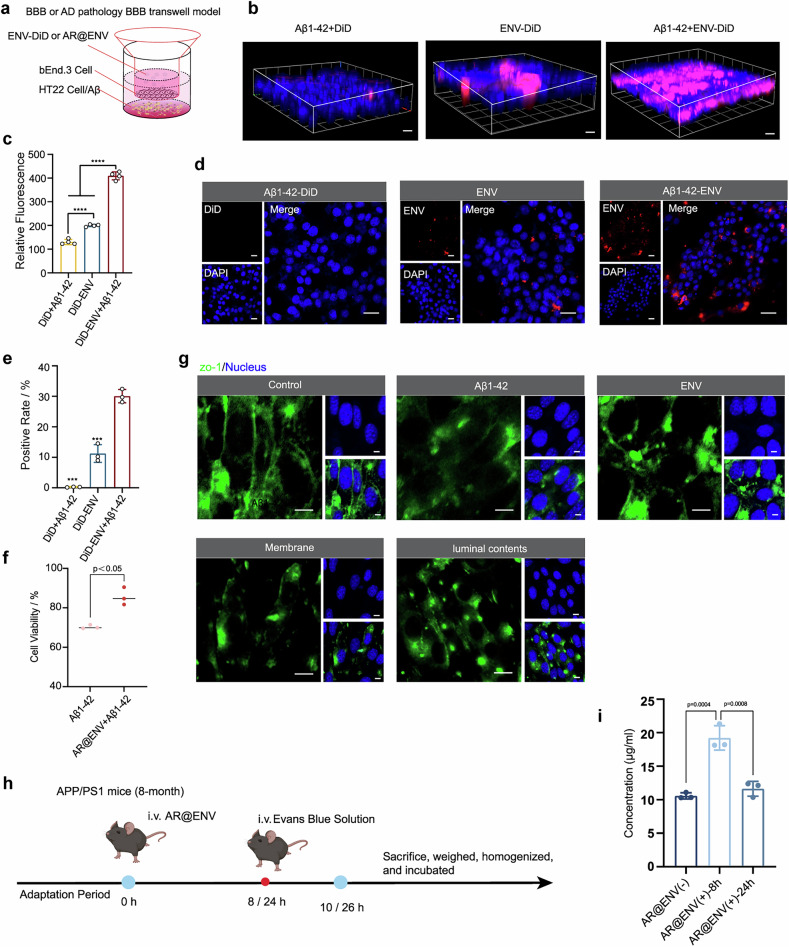
ENVs exhibit BBB crossing and neuronal targeting capabilities. **a** Scheme of the in vitro BBB or Aβ1‒42-induced AD BBB model established via a transwell assay. **b** Representative 3D confocal images of bEnd.3 cells following different treatments. Scale bars = 40 μm. **c** Fluorescence intensity measured in the lower chamber of the transwell model after different treatments. **d**, **e** Evaluation of ENV uptake by HT22 neuronal cells in the lower chamber of the transwell model after BBB traversal. **d** Representative confocal microscopy images. **e** Flow cytometry analysis of ENV uptake in neuronal cells. The data are presented as the means ± SDs compared with the ENV-DiD + Aβ1-42 group, *n* = 3. **f** Cytoprotective effect of AR@ENV against Aβ1‒42 oligomer-induced toxicity in neuronal cells following transwell traversal. The data are presented as the means ± SDs, *n* = 3. **g** Representative confocal microscopy images of ZO-1 protein expression in bEnd.3 cells after treatment. All scale bars = 20 μm. **h** Schematic diagram of the experimental protocol. Created with BioRender. **i** The concentration of Evans blue in the brain tissues of each group of mice. The data are presented as the means ± SDs, *n* = 3

To assess the transmembrane ability of ENVs, we established an in vitro BBB transwell model (Fig. 4a). bEnd.3 endothelial cells were cultured in the upper chamber, and BBB integrity was confirmed when the transendothelial electrical resistance (TEER) exceeded 200 Ω·cm².^35^ 3D confocal microscopy revealed that DiD-labeled ENVs (ENV-DiD) exhibited greater BBB penetration than free DiD did (Fig. 4b). Notably, ENV-DiD showed enhanced translocation in Aβ1‒42-induced AD BBB models, which was correlated with increased fluorescence intensity in the lower chamber (Fig. 4c). These findings suggest that ENVs more effectively traverse a compromised BBB, likely because of Aβ1‒42-induced inflammation, which alters BBB permeability.^36–38^ Given their microglial membrane properties, ENVs appear to exhibit superior transmigration across the disrupted BBB.

After crossing the BBB, we evaluated the uptake of ENV-DiD by HT22 neurons or microglia (BV2) in the lower chamber. Confocal microscopy further confirmed that ENV-DiD was internalized by the neuronal cells in the lower chamber (Fig. 4d; Supplementary Fig. 7b). Flow cytometry analysis revealed greater fluorescence intensity in the Aβ1–42-induced group than in the noninduced control and free DiD groups (Fig. 4e).

To determine whether drug-loaded AR@ENV exerts neuroprotective effects, we performed a CCK-8 assay. Aβ1-42 exposure reduced cell viability to less than 70%, but 24-hour AR@ENV treatment significantly restored viability in neurons (Fig. 4f; Supplementary Fig. 7c).

Furthermore, we investigated the mechanism of ENV translocation and examined the expression of ZO-1, a key tight junction protein, in bEnd.3 cells following ENV exposure. ENV treatment significantly reduced ZO-1 expression in the BBB model (Fig. 4g; Supplementary Fig. 8a, b). To determine whether this effect was mediated by surface proteins or internal contents, ENVs were fractionated into membrane components and luminal contents, which were then independently cocultured with bEnd.3 cells. Both the ENV membrane and luminal contents downregulated ZO-1 expression (Fig. 4g; Supplementary Fig. 8a, b), indicating that microglia-derived ENVs facilitate BBB transmigration via tight junction modulation.

Moreover, to assess the impact of AR@ENV on the integrity of the BBB in vivo, we utilized the Evans blue assay. The Evans blue assay is a widely recognized method for evaluating BBB integrity. The BBB, a highly selective semipermeable barrier, safeguards the brain from noxious substances in the bloodstream. When the BBB is disrupted, Evans blue, a large molecular dye, can infiltrate brain tissue. The quantification of Evans blue in brain tissue enables the assessment of BBB integrity. The experimental methods are illustrated in Fig. 4h. The results from the quantitative analysis revealed a significant increase in the Evans blue concentration within the brain tissues of the AR@ENV-injected mice at the 8 h time point relative to that of the control mice, which was indicative of potential BBB disruption. However, at 24 h, the concentration of Evans blue in the brain tissues did not significantly differ from that in the control group, suggesting that the disruption of the BBB elicited by AR@ENV is ephemeral and reversible (Fig. 4i).

An additional complex model was further established, which yielded results consistent with the aforementioned findings (Supplementary Fig. 9). These findings confirm that AR@ENV not only successfully crosses the BBB but also alleviates Aβ1-42-induced neurotoxicity, underscoring its potential as a therapeutic strategy for neurodegenerative disorders.

### In vivo biodistribution and brain targeting

**Fig. 5:**
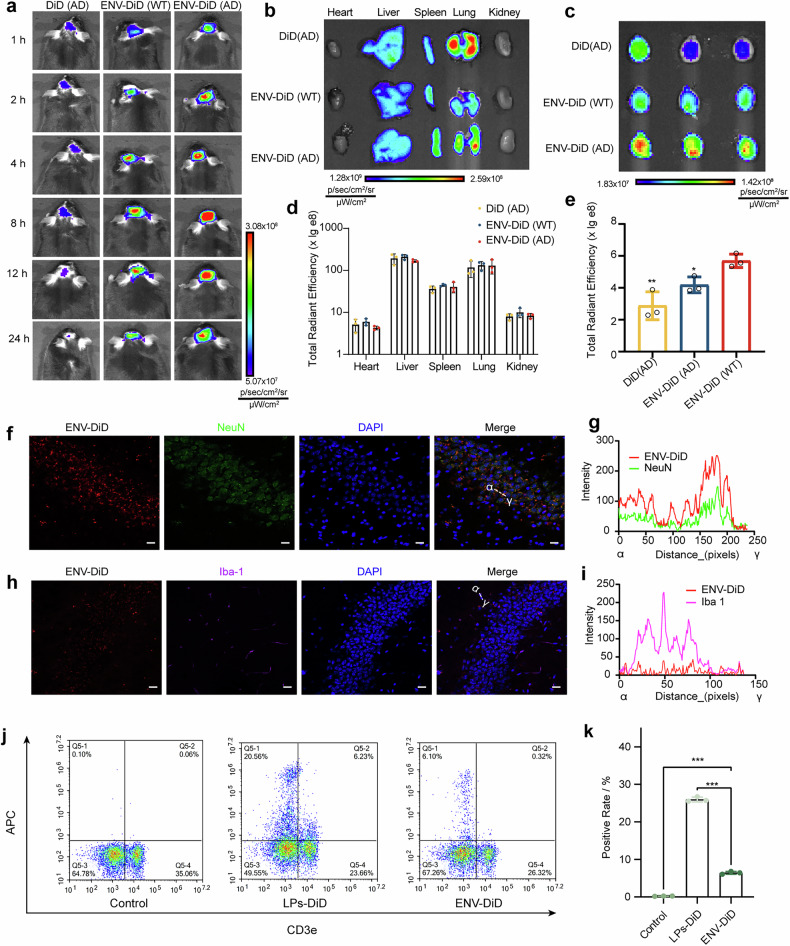
Biodistribution of ENV in APP/PS1 transgenic mice. **a** In vivo imaging of APP/PS1 or WT mice at specific time points after intravenous administration of DiD or ENV-DiD. Ex vivo imaging of major organs (**b**) and the brain (**c**). Semiquantitative analysis of total fluorescence intensity in major organs (**d**) and the brain (**e**). The data are presented as the means ± SDs and were compared to those of the ENV-DiD(AD) group, *n* = 3. **f** Immunofluorescence analysis of ENV-DiD (red) colocalization with neuronal markers (NeuN: green) in the APP/PS1 mouse brain, with DAPI staining for nuclei (blue). Scale bar: 20 μm. **g** Quantitative analysis of ENV-DiD and NeuN colocalization. **h** Immunofluorescence analysis of ENV-DiD (red) colocalization with microglial markers (Iba-1: purple) in the APP/PS1 mouse brain, with DAPI staining for nuclei (blue). Scale bar: 20 μm. **i** Quantitative analysis of ENV-DiD and Iba-1 colocalization. **j** Representative flow cytometry images of the uptake of ENV by lymphocytes in the blood. **k** Semiquantitative analysis of (**j**). The data are presented as the means ± SDs, *n* = 3

To assess the in vivo biodistribution and brain-targeting ability of ENVs, we evaluated their distribution in AD mouse models. Both APP/PS1 transgenic mice and an Aβ1‒42-induced AD models were used, with littermate controls and healthy Kunming mice used as comparative controls. DiD or DiD-labeled ENV (ENV-DiD) was administered via intravenous injection, and the fluorescence distribution was monitored over time via the Lumina III Imaging System. Compared with free DiD, ENV-DiD significantly improved brain targeting in AD mice, with increased BBB penetration in AD model mice compared with healthy controls (Fig. 5a and Supplementary Fig. 10a). At 24 h postadministration, ENV primarily accumulated in the liver, spleen, lung, and brain (Fig. 5b–e; Supplementary Fig. 10b, c). Notably, ENV-DiD resulted in the greatest degree of cerebral retention in AD mice, with a statistically significant increase compared with both control groups (Fig. 5c, e; Supplementary Fig. 10b, c).

To further validate these findings, major organs were collected and prepared into cryosections. Confocal microscopy confirmed that the fluorescence distributions in the tissues were consistent with the in vivo imaging results (Supplementary Fig. 11; Supplementary Fig. 12). The ENV is distributed mainly in the hippocampal region (Supplementary Fig. 13). Immunofluorescence staining revealed preferential ENV accumulation in neurons (Fig. 5f, g; Supplementary Fig. 14) rather than in microglia (Fig. 5h, I; Supplementary Fig. 14), suggesting a greater affinity for neuronal targeting. Furthermore, a minor fraction of ENV was also observed to be taken up by astrocytes (Supplementary Fig. 15). This may be attributed to the preservation of key receptor proteins, such as CX3CL1 and CD200, on the ENV surface, which interact with their respective neuronal ligands, CX3CR1 and CD200R, facilitating selective neuronal uptake.^39,40^

Given the susceptibility of nanodelivery systems to rapid clearance in vivo, the uptake of AR@ENV by immune cells and platelets was further investigated. Eight-month-old male APP/PS1 mice were administered ENV-DiD (DiD-labeled ENV substituting for the drug payload). At 8 h postinjection, the peak AR@ENV brain accumulation platelets and lymphocytes were isolated for flow cytometry analysis. The results demonstrated negligible uptake of ENV-DiD by platelets (Supplementary Fig. 16). Lymphocytes exhibited modest uptake of ENV-DiD, which was significantly lower than that of long-circulating liposomes. Furthermore, ENV-DiD uptake was predominantly localized to CD3E-positive lymphocytes (Fig. 5j, k).

To mimic the in vivo environment, the stability of rapamycin and AR7 within AR@ENV was assessed in 10% serum. Drug degradation was compared at 0 h and 8 h post-incubation. Approximately 12.8% degradation of rapamycin and 3.8% degradation of AR7 were detected after 8 h of serum exposure (Supplementary Fig. 17 and Supplementary Table 6).

These findings confirm the successful development of ENVs as BBB-penetrating nanovesicles, demonstrating efficient brain targeting in AD models.

### AR@ENV alleviated cognitive deficits in AD model mice

**Fig. 6:**
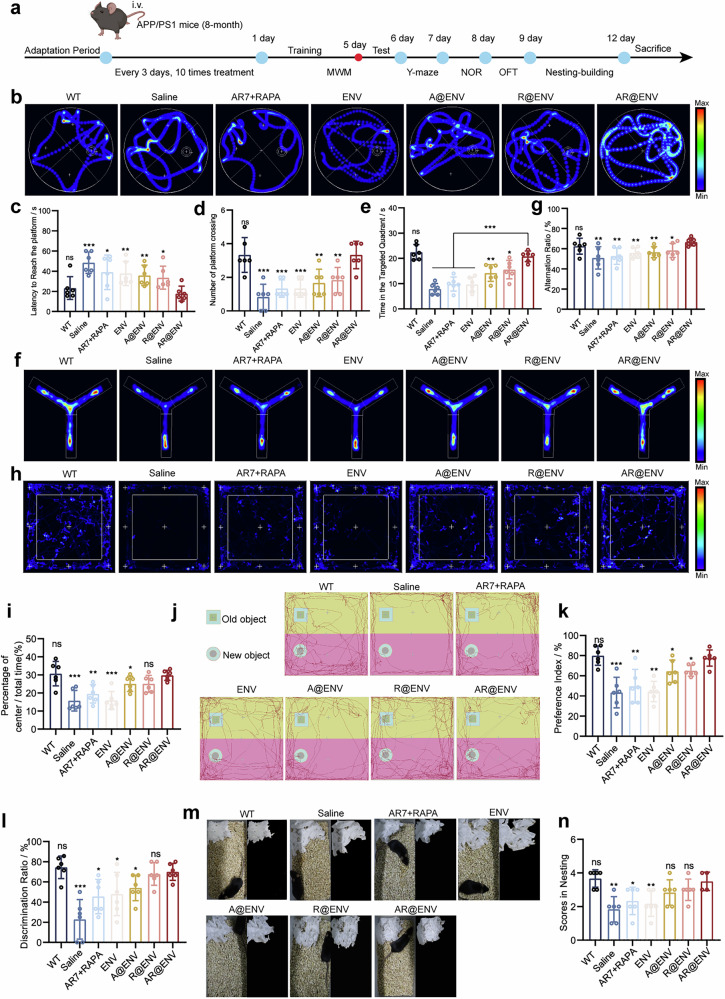
Behavioral evaluation of APP/PS1 mice following various treatments. **a** Schematic diagram of the experimental protocol. Created with BioRender. **b**–**e** Morris water maze (MWM) test for evaluating spatial learning and memory. **b** Representative heatmap of swimming path trajectories. **c** Latency to locate the target platform. **d** Number of platform crossings. **e** Time spent in the target quadrant. **f**, **g** Y-maze spontaneous alteration test assessing working memory. **f** Representative heatmap of movement patterns. **g** Spontaneous alternation percentage. **h**, **i** Open field test evaluating anxiety-like behavior: **h** Representative heatmap of movement trajectories. **i** Proportion of time spent in the central zone. **j**–**l** Novel object recognition (NOR) text assessing recognition memory: **j** Representative trajectory map. **k** Discrimination index. **l** Novel object preference index. **m**, **n** Nest-building test assessing cognitive and motor function: **m** Representative nest-building images. **n** Nest-building score. All the data are reported as the means ± SDs and were compared with those of the AR@ENV group, *n* = 6

We next evaluated the cognitive benefits of AR@ENV treatment in AD model mice. To assess its therapeutic potential, we conducted a comprehensive behavioral assessment focused on memory, spatial learning, and anxiety-related behaviors (Fig. 6a).

Compared with WT mice, APP/PS1 mice exhibited significantly prolonged escape latency in the Morris water maze (MWM) test, indicating that spatial learning and memory impairments are characteristic of AD (Fig. 6b, c). In contrast, AR@ENV-treated mice presented a marked reduction in escape latency, indicating an increased ability to locate the hidden platform. Moreover, AR@ENV treatment increased both the frequency of platform crossings and the time spent in the target quadrant, further confirming improvements in spatial memory and navigation skills (Fig. 6d, e). Similar cognitive improvements were observed in Aβ1-42-injected mice, reinforcing the broad applicability of AR@ENV across different AD models (Supplementary Fig. 18).

To further assess working memory and anxiety-related behaviors, we conducted Y-maze and open field tests. APP/PS1 mice exhibited a significant reduction in spontaneous alternations, indicating working memory deficits, whereas AR@ENV-treated mice restored alternation performance to WT-like levels, reflecting improved cognitive flexibility and spatial recognition (Fig. 6f, g). Additionally, in the open field test, APP/PS1 mice spent significantly less time in the central area, a behavior associated with heightened anxiety and cognitive decline (Fig. 6h, i). AR@ENV-treated mice presented a noticeable increase in center time, indicating reduced anxiety-like behavior and enhanced cognitive function.

To further validate cognitive restoration, we performed novel object recognition (NOR) and nest-building assays. In the NOR test, APP/PS1 mice exhibited reduced object recognition abilities, as indicated by their lower preference and discrimination indices. However, compared with WT mice, AR@ENV-treated mice presented a novel object recognition index and preference index, suggesting enhanced memory retention and cognitive restoration (Fig. 6j–l).

Similarly, in the nest-building assay, APP/PS1 mice and Aβ-42-injected mice exhibited impaired nest-building behavior, producing shallow, poorly structured nests (Fig. 6m; Supplementary Fig. 19a). In contrast, the AR@ENV-treated mice constructed well-formed nests with three-dimensional architecture and intact walls, similar to the WT mice (Fig. 6m). The higher nest-building scores in the AR@ENV group further support its role in restoring cognitive and motor functions (Fig. 6n and Supplementary Fig. 19b).

In addition to its cognitive benefits, AR@ENV also demonstrated superior safety compared with free drug administration. Hematological analysis revealed that free drug administration significantly reduced white blood cell (WBC) counts compared with those in WT mice (Supplementary Fig. 20a). Additionally, the saline and AR7+Rapa groups presented increased platelet (PLT) counts (Supplementary Fig. 20d). Importantly, biochemical indices revealed no significant abnormalities, suggesting that AR@ENV encapsulation effectively mitigated the systemic toxicity associated with free drug administration (Supplementary Fig. 21).

Taken together, these results demonstrate that AR@ENV significantly improves cognitive deficits in AD model mice by enhancing memory, reducing anxiety-like behaviors, and restoring cognitive function. Additionally, its encapsulated delivery system minimizes systemic toxicity, highlighting AR@ENV as a promising and safe therapeutic strategy for neurodegenerative disorders.

### AR@ENV increases autophagy and confers neuroprotection in AD mice

**Fig. 7:**
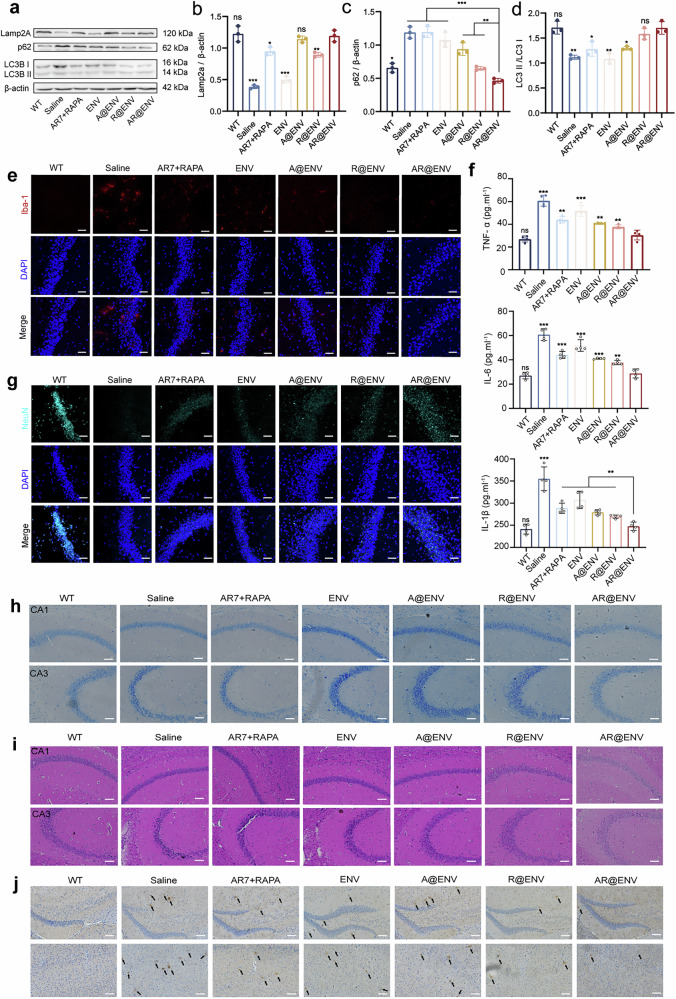
AR@ENV restores autophagy dynamics in the cerebral cortex of APP/PS1 mice, modulating the AD microenvironment and alleviating neurodegeneration. **a**–**d** Western blot analysis of CMA- and macroautophagy-associated protein expression in APP/PS1 mouse brains following different treatments. **a** Representative Western blot images. **b** Semiquantitative analysis of Lamp2A (CMA marker). **c** Semiquantitative analysis of p62. **d** Semiquantitative analysis of the LC3BII/LC3BI ratio (a macroautophagy marker). The data are presented as the means ± SDs and were compared with those of the AR@ENV group, *n* = 3. **e** Representative immunofluorescence images of activated microglia (Iba-1, red) across treatment groups. Nuclei were stained with DAPI (blue). Scale bar = 20 μm. **f** ELISA-based quantification of proinflammatory cytokines in the brain across different treatment groups. The data are presented as the means ± SDs and were compared with those of the AR@ENV group, *n* = 4. **g** Representative immunofluorescence images of NeuN (blue), indicating neuronal alterations in the brains of different treatment groups. Scale bar = 20 μm. **h** Nissl staining of brain sections from different treatment groups, including detailed images of the hippocampal CA1 and CA3 regions. Scale bars = 50 μm. **i** H&E staining of brain sections from different treatment groups, highlighting the hippocampal CA1 and CA3 regions. Scale bars = 50 μm. **j** Immunohistochemical staining of Aβ plaques, indicated by arrows, in the brains of various treatment groups

Given the cognitive improvements observed in AR@ENV-treated mice, we next investigated the therapeutic effects of AR@ENV at the molecular and cellular levels, with a focus on autophagy restoration and neuroprotection in the AD brain. Since autophagy dysfunction is a hallmark of AD, we examined the expression of autophagy-related proteins (Fig. 7a; Supplementary Figs. 22 and 23). AR@ENV treatment significantly upregulated Lamp2A, a key regulator of the CMA pathway (Fig. 7b; Supplementary Fig. 22b), while increasing the LC3BII/LC3BI ratio and reducing p62 levels, indicative of macroautophagy activation (Fig. 7c, d; Supplementary Fig. 22c, d). Interestingly, R@ENV treatment upregulated Lamp2A, whereas A@ENV treatment primarily enhanced CMA (Fig. 7b; Supplementary Fig. 22b), suggesting that restoring autophagic activity in neurons may promote broader cellular homeostasis.

Since neuroinflammation is a major driver of AD progression, we next examined the effects of AR@ENV on the microglial response and brain inflammation. In AD, microglia accumulate around Aβ plaques and shift between proinflammatory (M1, neurotoxic) and anti-inflammatory (M2, neuroprotective) states.^41^ Confocal microscopy revealed a significant increase in M1 microglia in saline-treated AD mice, contributing to widespread neuroinflammation (Fig. 7e; Supplementary Fig. 24). However, AR@ENV treatment significantly reduced M1 microglial activation, restoring the levels to those observed in WT mice, indicating that AR@ENV modulates microglial activation to suppress neuroinflammation.

Reactive astrogliosis promotes Aβ deposition and generates neurotoxic byproducts (e.g., putrescine, ammonia, H₂O₂) via the urea cycle, contributing to neuronal damage and memory impairment. Consequently, the effect of AR@ENV treatment on astrogliosis was examined. Compared with wild-type (WT) mice, APP/PS1 mice exhibited significant astrogliosis in brain tissue. AR@ENV intervention effectively ameliorated this pathological proliferation (Supplementary Fig. 25).

To further confirm its anti-inflammatory effects, we quantified the proinflammatory cytokines TNF-α, IL-6, and IL-1β in AD mouse brains via ELISA (Fig. 7f; Supplementary Fig. 26). AR@ENV treatment significantly reduced all three inflammatory markers, confirming that it effectively suppresses neuroinflammation and improves the AD brain microenvironment.

Since neuronal loss is correlated with memory deficits in AD, we next examined neuronal survival in the hippocampal CA1 and CA3 regions, areas crucial for learning and memory.^42,43^ Immunofluorescence staining revealed that AR@ENV-treated mice had significantly more NeuN^+^ neurons than saline-treated AD mice did (Fig. 7g; Supplementary Fig. 27), indicating substantial neuroprotection and reduced neuronal loss.

These findings were further supported by Nissl staining, where AR@ENV-treated mice presented well-preserved Nissl bodies and intact neuronal structures, with no significant difference from those of WT mice (Fig. 7h; Supplementary Figs. 28, 30). Similarly, H&E staining of saline-treated AD mice revealed severe neurodegeneration characterized by pyramidal cell necrosis, nuclear pyknosis, and widespread neuronal damage (Fig. 7i; Supplementary Figs. 29, 30). AR@ENV treatment significantly reversed these degenerative changes, restoring normal hippocampal morphology.

Since Aβ accumulation is a hallmark of AD, we next examined whether AR@ENV reduces the Aβ burden in the APP/PS1 mouse brain. AR@ENV-treated mice presented a marked reduction in Aβ plaque deposition, particularly in the hippocampus (Fig. 7j). These findings indicate that AR@ENV not only protects neurons but also enhances the clearance of neurotoxic Aβ aggregates.

Taken together, these results demonstrate that AR@ENV enhances autophagy, reduces neuroinflammation, preserves neuronal integrity, and promotes Aβ clearance in AD model mice. By restoring autophagic flux and modulating the inflammatory microenvironment, AR@ENV confers neuroprotection and represents a promising therapeutic strategy for AD.

### Biocompatibility of AR@ENV

Given the therapeutic efficacy of AR@ENV in ameliorating AD pathology, we next evaluated its biocompatibility and systemic safety to assess its suitability for in vivo applications. Hemolysis assays demonstrated that AR@ENV exhibited minimal hemolytic activity, with hemolysis rates remaining below 5% across various concentrations within 8 h. Erythrocytes retained a normal morphology without signs of crenation or rupture, indicating a negligible risk of red blood cell damage (Supplementary Fig. 31a–c).

We further assessed cytotoxicity in vitro in HT22 cells. Four types of nanovesicles were tested: ENV, A@ENV, R@ENV, and AR@ENV. After 24 h of treatment, the cell viability in all the groups exceeded 80%, confirming favorable biocompatibility (Supplementary Fig. 32a).

To evaluate systemic safety in vivo, healthy Kunming mice were treated daily with seven doses of saline, free AR7+RAPA, ENV, or AR@ENV. H&E staining of major organs revealed no tissue lesions in any group (Supplementary Fig. 32b), supporting the absence of organ toxicity. Hematological parameters remained within normal ranges across all treatments (Supplementary Fig. 32c), and biochemical analysis revealed that AR@ENV significantly reduced the hepatotoxicity observed with free drug administration (Supplementary Fig. 32d). Additionally, AR@ENV treatment led to a significant reduction in circulating low-density lipoprotein (LDL) levels, suggesting potential metabolic benefits. The body weights are shown in the supplementary table. across the study (Supplementary Fig. 32e), further supporting systemic safety.

To evaluate potential immune responses, we quantified the inflammatory cytokines TNF-α, IL-6, and IL-1β in both serum and brain tissue after treatment. No significant elevation in cytokine levels was observed in any group, indicating that AR@ENV does not elicit measurable immunogenicity or proinflammatory responses (Supplementary Fig. 32f).

To systematically evaluate the in vivo immune compatibility of AR@ENV, Kunming mice (6–8 weeks old) received ten intravenous tail vein administrations every 3 days. Serum and spleens were collected 7 days after the final drug administration. ELISAs revealed no significant differences in total IgG, IgG1, or IgG2a levels versus controls, indicating the absence of humoral immune activation (Supplementary Fig. 33). Flow cytometric analysis further demonstrated that while AR@ENV did not alter CD4⁺ T-cell populations (Supplementary Fig. 34a, c), it significantly increased the proportion of CD8⁺ T cells (Supplementary Fig. 3b, c). Given the documented evidence that CD8⁺ T cells mitigate AD progression by suppressing hyperactivated microglia, this immunomodulatory effect may provide complementary therapeutic synergy to AR@ENV’s primary efficacy.

## Discussion

Alzheimer’s disease (AD) remains a formidable neurodegenerative disorder with limited therapeutic options. Current FDA-approved drugs, including acetylcholinesterase inhibitors (e.g., donepezil) and NMDA antagonists (e.g., memantine), provide symptomatic relief but fail to halt disease progression.^7^ Second-generation anti-Aβ monoclonal antibodies (mAbs), such as Lecanemab and Donanemab, demonstrate efficacy in reducing amyloid plaques but face significant limitations: (1) increased risk of amyloid-related imaging abnormalities (ARIA, including edema/effusions and microhemorrhages), which occur in 12.5–36.8% of patients in clinical trials;^8^ (2) poor blood-brain barrier (BBB) penetration, limiting central nervous system exposure;^9^ and (3) inability to prevent new Aβ deposition or tackle tau pathology. ^11^Tau-targeting strategies (e.g., tau aggregation inhibitors) have similarly encountered clinical obstacles due to insufficient target engagement or off-target effects. Critically, both Aβ and tau aggregates drive neurodegeneration through multifaceted pathways, including protein misfolding, neuroinflammation, and proteostasis disruption.^3^ Among these, impaired autophagy emerges as a pivotal node in pathogenic cascades, as it compromises cellular clearance mechanisms, leading to toxic protein accumulation.^44^ Macroautophagy (degrading Aβ and pathological tau via ATG5/SIRT1/PPARα pathways) and chaperone-mediated autophagy (CMA, regulating APP processing and soluble tau homeostasis) represent complementary pathways whose synchronous activation could offer a dual-mechanism strategy—restricting nascent neurotoxic protein production while accelerating aggregate degradation.^18,45^

Our study provides compelling evidence that both macroautophagy and chaperone-mediated autophagy (CMA) are significantly impaired in the brains of APP/PS1 mice, with in vitro experiments demonstrating that Aβ1–42 oligomers selectively suppress macroautophagy without altering CMA markers. These findings substantiate the growing consensus that autophagy dysfunction is not merely a downstream consequence of Aβ toxicity but represents an early pathological event in Alzheimer’s disease (AD), consistent with previous observations that autophagic disruption precedes Aβ deposition.^14^ The ability of Aβ1–42 oligomers to exacerbate macroautophagy decline underscores a vicious pathological feedback loop wherein impaired protein clearance and toxic protein accumulation mutually reinforce each other. Given the early involvement of autophagy dysfunction in AD pathogenesis, autophagy-related molecules (e.g., LC3, p62, and LAMP-2A) emerge as promising preclinical biomarkers that could facilitate earlier diagnosis and intervention. While established biomarkers such as Aβ, total tau, phosphorylated tau (Thr181), and neurofilament light chain (NFL) primarily reflect later-stage pathology,^46–48^ autophagy signatures may offer a critical window for therapeutic intervention at preclinical stages, potentially enabling disease-modifying strategies that target fundamental proteostatic mechanisms before irreversible neurodegeneration occurs.

To restore both autophagy pathways synchronously, we engineered microglia-derived nanovehicles (AR@ENVs) via the MiLi-FE (microfluidic lipid extrusion) process for the codelivery of the macroautophagy inducer rapamycin and the CMA activator AR7. This method enables efficient vesicle production while preserving key membrane properties. Notably, AR@ENV displayed superior BBB penetration both in vitro and in vivo (Figs. 4 and 5), which was facilitated in part by its capacity to downregulate tight junction proteins such as ZO-1 on endothelial cells, a mechanism that may explain its enhanced brain translocation. Once AR@ENV crosses the BBB, it preferentially accumulates in neurons rather than in microglia (Fig. 5), likely due to specific membrane protein interactions (e.g., CX3CL1-CX3CR1 and CD200-CD200R).^40^ This selective targeting echoes findings from prior studies showing that exosome-like vesicles exhibit neuron-specific tropism in AD models,^49^ although the full mechanism remains to be elucidated. Functionally, rapamycin-induced macroautophagy facilitated Aβ degradation and alleviated oxidative stress and neuroinflammation, whereas AR7-mediated CMA selectively cleared misfolded proteins and restored proteostasis. The synergy between these pathways contributed to significant cognitive improvements in both APP/PS1- and Aβ-injected AD mice (Fig. 6). Histological analyses further revealed attenuation of neuronal degeneration and reduced neuroinflammation following AR@ENV treatment (Fig. 7). These neuroprotective effects are likely mediated by the restoration of autophagic flux, suppression of pathological microglial activation, and modulation of the neuroinflammatory microenvironment.^50^ Meanwhile, this study conducted a systematic in vivo safety evaluation of the AR@ENV. Short-term repeated administration experiments demonstrated that AR@ENV did not induce abnormal body weight changes in mice, nor did it elevate inflammatory cytokine (TNF-α, IL-6, IL-1β) levels in serum or brain tissues (Supplementary Fig. 32). Major organs showed no pathological lesions, and hematological parameters remained within normal ranges. Notably, AR@ENV effectively prevented the liver injury potentially induced by free drugs and significantly reduced low-density lipoprotein levels (Supplementary Fig. 32). In terms of immunogenicity, no significant changes in specific antibody levels (IgG/IgG1/IgG2a) were detected after long-term administration, indicating no evident humoral immune response (Supplementary Figs. 33, 34). Although AR@ENV did not affect CD4+ T cell counts, it significantly increased CD8+ T cell levels. Existing studies suggest that CD8+ T cells can mitigate Alzheimer’s disease progression by modulating microglial function, implying that this observed effect may offer potential therapeutic benefits.^51^ In conclusion, AR@ENV demonstrates favorable biosafety profiles.

While these results underscore the promising therapeutic profile of AR@ENV, several key translational challenges must be systematically addressed before clinical application can be realized. First, the precise mechanisms underlying the traversal of microglia-derived nanovesicles across the blood-brain barrier and their selective homing to disease-afflicted brain regions remain incompletely elucidated. A deeper understanding of the molecular determinants—such as specific receptor-ligand interactions and transient modulation of BBB integrity via proteins like ZO-1—is essential for rational vehicle optimization. Furthermore, refining critical pharmaceutical properties, including drug loading efficiency, vesicle stability under physiological conditions, and long-term biosafety, will be imperative for clinical viability. To enhance targeting precision and pharmacokinetic performance, future work should prioritize advanced bioengineering strategies such as surface functionalization with neuron-specific ligands (e.g., RVG peptides or transferrin receptors) or modulation of vesicle membrane composition to improve circulatory half-life and reduce immunogenicity. Additionally, comprehensive toxicological evaluations and scale-up production processes must be established to ensure that AR@ENV meets regulatory standards for eventual human trials.

In summary, this study presents a strategy for the synchronized activation of macroautophagy and CMA to restore proteostasis and cognitive function in AD. We developed AR@ENV via the MiLi-FE process, which enables scalable production of microglia-derived nanocarriers coloaded with rapamycin and AR7. AR@ENV effectively crossed the BBB, targeted neurons, mitigated neuroinflammation, and significantly improved cognitive deficits in AD model mice. In addition to AD, the MiLi-FE platform provides a versatile system for engineering cell-derived nanovesicles, offering broad potential for treating neurodegenerative diseases characterized by autophagy disruption and protein aggregation. By addressing autophagy dysregulation at multiple levels, this approach represents a promising direction for next-generation therapeutics in AD and related disorders.

## Materials and methods

### Materials

DSPE-PEG2000 was purchased from Hunan Huateng Biological Technology (China). The Cell Counting Kit-8 (CCK-8) and BCA protein assay kits were purchased from Beyotime Biotechnology Co., Ltd. (Shanghai, China). Mouse IL-6, IL-1β, and TNF-α ELISA kits were purchased from Thermo Fisher Scientific (USA). AR7 and rapamycin were purchased from MedChemExpress (USA). Mouse Lamp2A, NeuN, Iba-1 and CX3CR1 antibodies were purchased from Abcam (USA). LC3B, p62, and GAPDH antibodies were purchased from Abways (Shanghai, China). CD9, TSG101, PINK1, and Parkin antibodies were purchased from HUABIO (Hangzhou, China). IgG, IgG1 and IgG2a were purchased from ABclonal (Wuhan, China). Aβ1-42 was purchased from Sangon Biotech (Shanghai, China).

### Cells and animals

All chemicals used were of analytical or reagent grade. Mouse brain microvascular endothelial cells (bEnd.3), mouse hippocampal neuronal cells (HT22) and a murine microglial cell line (BV2) were obtained from the Cell Bank of the Chinese Academy of Sciences (Shanghai, China). Male Kunming mice (6–8 weeks old) were obtained from Chengdu Dashuo (Chengdu, China). Male APP/PS1 mice (8 months) were obtained from Changzhou Cavens (Changzhou, China). All animal experiments were approved by the ethics committee of Sichuan University.

### Preparation of AR7@LP and Rapa@LP

The preparation of AR7 and rapamycin liposomes (LPs) followed previously established methods. Briefly, AR7 or rapamycin was dissolved with soybean phospholipids, cholesterol, and DSPE-PEG-2000 (in a 3:1:1 mass ratio), and each component was separately dissolved in dichloromethane. The drug-to-lipid ratio was either 40:1 or 20:1. Liposomes are prepared via the thin-film hydration method, followed by probe sonication after hydration to obtain the desired liposomes.

### Preparation of liposomes colabeled with FITC and DiD

The specific methodology employed for coencapsulating lipophilic DiD and hydrophilic FITC into liposomes was the thin-film hydration-extrusion method, which was chosen for its reliability and controllability. This involved first dissolving DiD, soybean phospholipid, cholesterol, and DSPE-PEG-2000 (at a mass ratio of 3:1:1) in dichloromethane, with a DiD-to-lipid mass ratio of 1:100. The mixture was then rotary evaporated at 40 °C to form a homogeneous thin film. The lipid film was subsequently hydrated via a buffer solution containing 0.1 mg/mL FITC, with hydration assisted by probe sonication at 80 W for 2 min. Finally, the suspension was extruded 5 times through a 200 nm polycarbonate membrane to yield liposomes coloaded with both DiD and FITC.

### Optimization of critical parameters for liposome internalization in BV2 microglia

(1) BV2 cells were seeded in 96-well plates and cultured to 50–60% confluence. The medium was then replaced with liposomes loaded with AR7 and rapamycin. Cell viability was assessed via the CCK-8 assay at 2 h, 4 h, 6 h, 8 h, 12 h, and 24 h posttreatment. (2) BV2 cells were seeded in 24-well plates and cultured to 50–60% confluence. After treatment with DiD-loaded liposomes for 2 h, 4 h, 6 h, or 8 h, the lysosomes were stained with a LysoTracker probe. The cells were fixed with 4% paraformaldehyde, the nuclei were counterstained with DAPI, and mounted slides were imaged via confocal microscopy to evaluate liposome‒lysosome colocalization. (3) BV2 cells were seeded in 6-well plates and cultured to 50–60% confluence. Following treatment with AR7/rapamycin-loaded liposomes for 2 h, 4 h, 6 h, or 8 h, the cells were harvested and lysed with acetonitrile to extract AR7 and rapamycin. After centrifugation to remove the precipitates, the drug concentrations were quantified via HPLC.

### Preparation of AR@ENV

The preparation of AR@ENV was modified on the basis of the literature.^52^ First, BV2 cells were cultured to 80% confluence and then treated with AR7@LP (containing 30 μg/mL AR7) or Rapa@LP (containing 10 μg/mL rapamycin) for 8 h. The cells were collected and extruded through polycarbonate membranes with pore sizes of 5 µm, 0.8 µm, 0.4 µm, and 0.1 µm (each size was passed through three times). The precipitate was subsequently collected by ultracentrifugation at 1,000,000 × *g*, and the resulting AR@ENV was stored at −80 °C.

### In vitro simulation of the degradation profile of AR@ENV under physiological conditions

To simulate the degradation of AR@ENV in vitro, the samples were incubated in PBS containing 10% FBS at 37 °C with shaking (60 rpm/min) for 8 h. Acetonitrile was added to lyse the vesicles, and rapamycin and AR7 were extracted. The drug content was quantified by HPLC, with the 0-h incubation group serving as the control. The degradation profiles of rapamycin and AR7 were calculated on the basis of residual drug content.

### Characterization of AR7@ENV

The distinctive proteins TSG101 and CD9, which are characteristic of vesicles, and the microglia-specific receptor CX3CR1 were identified through Western blotting (WB). Moreover, comprehensive proteomic profiling was conducted to analyze the entire proteome of BV2 cells, BV2 plasma membranes, and extracellular vesicles (ENVs). The morphology of the native vesicles, ENVs, and AR@ENVs was observed via transmission electron microscopy (TEM), and nanoparticle tracking analysis (NTA) was used to measure the particle size, Span and zeta potential of the vesicles. To verify whether the liposomes disperse after being taken up, DID and FITC were coencapsulated within the liposomes, and the colocalization results were used.

### Cell viability assay

AR@ENV viability was evaluated through the CCK8 assay. HT22 and BV22 cells were seeded in 96-well plates and cultured to 50–60% confluence prior to treatment with ENV, A@ENV, R@ENV, or AR@ENV at defined concentrations: total vesicle protein concentrations of 10, 20, 50, and 100 μg/mL, corresponding to rapamycin loads of 0.07, 0.14, 0.36, and 0.72 μg/mL and AR7 loads of 0.20, 0.40, 1.00, and 2.00 μg/mL. After a 24-h incubation, the medium was replaced, and 10 µL of CCK8 reagent was added to each well. The cells were incubated until a color change occurred, and the absorbance was measured at 450 nm.

### Anti-Aβ1-42 toxicity assay

HT22 cells were inoculated onto 96-well plates and cultured for 24 h. The original medium was then aspirated and replaced with medium containing Aβ1-42 at a concentration of 15 μM. The cells were subsequently treated with ENV, A@ENV, R@ENV, or AR@ENV at the following concentrations: total vesicle protein at 1, 2, 5, and 10 μg/mL; corresponding rapamycin concentrations of 7, 14, 35, and 70 ng/mL; and AR7 concentrations of 20, 40, 100, and 200 ng/ml. After a 24-h incubation period, the medium was changed, and 10% CCK8 reagent was added to each well. The plates were then incubated until a color change was observed, after which the absorbance was measured at a wavelength of 450 nm.

### In vitro blood‒brain barrier (BBB) model assay

bEnd.3 cells were seeded at a density of 1 × 10^5^ cells per well in the upper chambers (12-well, pore size: 3 μm; Corning, USA). The culture medium was replenished daily, and the transendothelial electrical resistance (TEER) values were measured until they reached a value of 200 Ω/cm^2^. Subsequently, HT22 cells were seeded in the lower chambers and induced with fresh culture medium containing Aβ1-42 (15 μM) for 24 h. Next, the upper chamber was treated with DiD-labeled ENV at a specified concentration. After incubation for 4 h, the fluorescence intensity in the culture medium of each lower chamber was quantified via a microplate reader. The average fluorescence intensity in HT22 cells was assessed through flow cytometry, and the penetration of DiD-ENV through the bEnd.3 monolayer was examined via 3D confocal microscopy.

### Western blotting analysis

Western blotting was performed following methods described in the literature.^53^ Briefly, cells or tissues were collected posttreatment. The cells or tissues were lysed via lysis buffer, with additional homogenization of the tissues. The obtained protein lysates were quantified via a BCA assay kit. The proteins were subsequently denatured by heating at 100 °C for 10 minutes. The proteins were separated via 10% SDS‒polyacrylamide gel electrophoresis (SDS‒PAGE) and then electrophoretically transferred onto polyvinylidene fluoride (PVDF) membranes. The membranes were blocked with 5% skim milk at room temperature for 2 hours, followed by an overnight incubation with specific primary antibodies at 4 °C. Afterward, the membranes were incubated with secondary antibodies at room temperature for 1 h. Finally, the protein bands were visualized via a gel imaging system.

### Effect of AR@ENV on the mTOR pathway assessed by qRT‒PCR

To assess the impact of AR@ENV on the mTOR pathway via qRT‒PCR, HT22 cells were seeded in 12-well plates and pretreated with 15 μM Aβ1‒42 for 12 h. Cells were then exposed to ENV or AR@ENV at specified concentrations: 5 μg/mL total vesicle protein, 35 ng/mL rapamycin, and 100 ng/mL AR7. Untreated cells (without Aβ1‒42) and Aβ1‒42-only treatment were used as control groups. After 12 h of incubation, total RNA was extracted, and qRT‒PCR was performed to quantify the expression of core mTOR pathway genes (Mtor, Akt1, and Pten).

### Construction of the AD animal model

Amyloid-β1-42 (Aβ1-42) was dissolved in artificial cerebrospinal fluid (CSF) at a concentration of 1 mg/mL and preincubated at 37 °C for 7 days. Male Kunming mice were subsequently anesthetized and mounted in a stereotactic frame. Aβ1-42 (5 μL) was injected into the right hippocampus of Kunming mice, and the injection needle was slowly withdrawn 8 minutes post-injection.

### In vivo distribution study

APP/PS1 mice and Aβ-injected model mice were randomly assigned to different groups, with littermate control mice and healthy Kunming mice serving as the control groups. Subsequently, free DiD or DiD-labeled ENV (DiD dosage of 10 mg/kg) was administered, and imaging was performed at specific time points of 1, 2, 4, 8, 12, and 24 h postadministration via the Lumina III Imaging System (PerkinElmer, USA). At the designated time points, the mice were euthanized for tissue collection, which was then used for ex vivo imaging. The tissues were fixed and dehydrated, followed by sectioning via a freezing microtome (Leica CM1950, Germany). The sections were subjected to immunofluorescence staining, and after counterstaining with DAPI, the slides were mounted. The slides were then observed under a confocal microscope.

### Systemic clearance assay of AR@ENV

Eight-month-old male APP/PS1 mice were administered DiD-labeled ENVs (ENV-DiD) as a drug surrogate. At 8 h postinjection, blood was collected via retro-orbital puncture into EDTA-anticoagulated tubes and centrifuged at 800 rpm/min for 15 min. The upper plasma layer was transferred to a new tube and centrifuged at 3500 rpm/min for 10 min to obtain a platelet-rich pellet. Separately, whole blood was treated with lymphocyte separation medium for lymphocyte isolation according to the manufacturer’s protocol. Uptake of ENV-DiD by lymphocytes and platelets was quantified via flow cytometry.

### AD treatment and testing protocol

APP/PS1 mice and Aβ-injected mice were randomly assigned to six groups, each receiving intravenous injections of either saline (APP/PS1 group), free drug (AR7+Rapa), ENV, A@ENV, R@ENV, or AR@ENV. The dosing concentrations are ~0.2 mg/kg for rapamycin, 0.6 mg/kg for the agent AR, and 30.0 mg/kg for vesicular carrier proteins.^54^ Injections were administered every 3 days for a total of 10 times. C57BL/6 mice or healthy Kunming mice served as the wild-type (WT) control groups and were injected with saline. Body weight was recorded every 3 days. After completion of the treatment, behavioral experiments were conducted, and the mice were euthanized for further research.

### Morris water maze test

The Morris water maze test is a widely used behavioral assay in neuroscience for evaluating spatial learning and memory in mice. The experiment involved a circular pool divided into four invisible quadrants, with a platform hidden 1 cm below the water surface in one of the quadrants. Various colored cues—namely, a triangle, a circle, a square, and a pentagon made from construction paper—were placed around the pool to provide spatial reference points. The water was made opaque with a small amount of titanium dioxide, and its temperature was maintained at 22 ± 1 °C. An overhead video tracking system recorded the swimming trajectories of the mice. The testing period spanned 6 days and included a hidden platform test (days 1–5) and a probe test (day 6). During the hidden platform test, the mice were placed randomly in each quadrant of the pool and were given 60 s to locate the hidden platform. Testing occurred daily in three different quadrants, with a 4-h interval between trials, and escape latencies were recorded. On the final day, a 24-h probe test was conducted with the platform removed, allowing the mice to swim freely for 60 s. The video tracking system captured their trajectories, and the data were analyzed to assess spatial learning and memory by examining escape latencies and swimming speeds.

### Y-Maze test

The Y-Maze test is used to assess spatial working memory and cognitive flexibility in mice. The maze consists of three arms of equal length, typically colored black or blue, and intersects at a central point to form a Y shape. During the experiment, the apparatus was placed in a quiet testing room with constant lighting, and a camera tracking system was used to monitor the movements of the mice. At the start of the test, the mouse was placed at the end of one arm, facing the end of the arm, and was allowed to freely explore the maze for 8 minutes. The trajectory of each mouse was recorded throughout the experiment.

### Open field test (OFT)

Prior to the experiment, the testing chamber was cleaned, and the environment was quiet with uniform lighting. During the experiment, the animal was placed in the center of the chamber, the experimenter quickly left, and behavior analysis software was used to record data on the animal’s movement trajectory, velocity, dwell time, and other parameters for 10 min.

### Novel object recognition (NOR) test

Before the experiment, the mice were allowed to acclimate to the arena for 10 min. During the familiarization phase, they explored two identical objects (Object A and Object A) for 10 min. In the testing phase, one object was replaced with a novel object (Object B), and the mice explored the arena for an additional 10 min. The time spent by the mice exploring the novel object (Object B) and the familiar object (Object A) was recorded.

### Nest-building test

Before the experiment begins, the mice should be acclimated to the environment to minimize the impact of environmental factors on the results. The mice were subsequently placed in prepared cages, and an adequate amount of nesting material was provided. We observed and recorded how the mice used these materials to construct nests over the following 72 h. When evaluating nesting behavior, the focus is on the structure, comfort, and integrity of nests to gain a comprehensive understanding of their quality and complexity.

### Evans blue assay for blood‒brain barrier permeability

Eight-month-old male APP/PS1 mice received intravenous tail vein injections of AR@ENV at therapeutic concentrations. Age-matched APP/PS1 mice without AR@ENV injection served as blank controls. At 8 h and 24 h postinjection (time points corresponding to peak cerebral accumulation of AR@ENV as determined by distribution studies), 2% Evans blue solution (2 ml/kg) was administered via the tail vein. After 2 h, the mice were euthanized and subjected to transcardiac perfusion: the thoracic cavity was surgically exposed, and heparinized 0.9% saline was slowly infused through the cardiac apex until the effluent from the incised right atrium became clear. The brain tissues were then harvested, weighed, and homogenized. The tissue samples were incubated in formamide (100 mg of tissue per 1 ml of formamide) at 37 °C with 80 rpm orbital shaking for 48 h. The supernatants were subsequently analyzed via spectrophotometry at 620 nm.

### In vivo safety assessment of AR@ENV

Kunming mice were randomized into four treatment groups: saline control, AR7 + Rapa, ENV, and AR@ENV. Intravenous administration was performed every other day for seven doses at approximate concentrations of 0.2 mg/kg rapamycin, 0.6 mg/kg AR7, and 30.0 mg/kg vesicular carrier protein across all drug-treated cohorts. Body weight fluctuations were recorded throughout the intervention period. The final procedures included the collection of major organs for hematoxylin and eosin (H&E) histopathological examination, alongside the procurement of whole blood for complete hemogram analysis and serum for biochemical profiling and cytokine quantification.

### In vivo immunogenicity assessment of AR@ENV

Healthy Kunming mice were randomized into two groups receiving intravenous injections of either saline (control) or AR@ENV nanocomplexes at approximate concentrations of 0.2 mg/kg rapamycin, 0.6 mg/kg AR7, or 30.0 mg/kg vesicular carrier protein. The drugs were administered every 72 h for a total of 10 doses. Posttreatment, serum and spleen samples were collected. Serum samples were subjected to ELISA to quantify antigen-specific antibody titers (IgG, IgG1, and IgG2a), while splenocytes were isolated via mechanical dissociation for flow cytometric quantification of CD4⁺ and CD8⁺ T-cell populations.

### Statistical analysis

All the experiments were performed via GraphPad Prism 9. All experiments were repeated at least three times. The data are presented as the means ± standard deviations (SDs). Statistical comparisons were performed via two-tailed *t* tests and one-way ANOVA. A *p* value < 0.05 was considered significant.

## Supplementary information

Supporting information

Uncropped blot

## Data Availability

All data supporting the findings of this study are available within the article and its Supplementary Information.
