# Supplementary material for: Microglia-derived nanovesicles synchronize macroautophagy and chaperone-mediated autophagy for Alzheimer’s disease therapy
Source: Signal Transduct Target Ther. 2025 Nov 3;10:360. doi: 10.1038/s41392-025-02453-y (PMC12583606; doi:10.1038/s41392-025-02453-y)
Supplement: Supplementary file 1 — Supporting information [file 41392_2025_2453_MOESM1_ESM.pdf]

Supporting Information for

Microglia-derived nanovesicles synchronize macroautophagy and chaperone-mediated autophagy for alzheimer's therapy

Min Li, Shuang Chen, Rong Guo, Yang Wang, Mingrui Yang, Yingke Liu, Shiyu Zhu, Qiang Zhang, Jiaxin Li, Fang Chen, Bo Wang, Man Li, Qin He.

Correspondence to: qinhe317@126.com or manli@scu.edu.cn

This PDF file includes:

Supplementary Figures 1 to 34

Supplementary Tables 1 to 8

Supplementary Figure 1

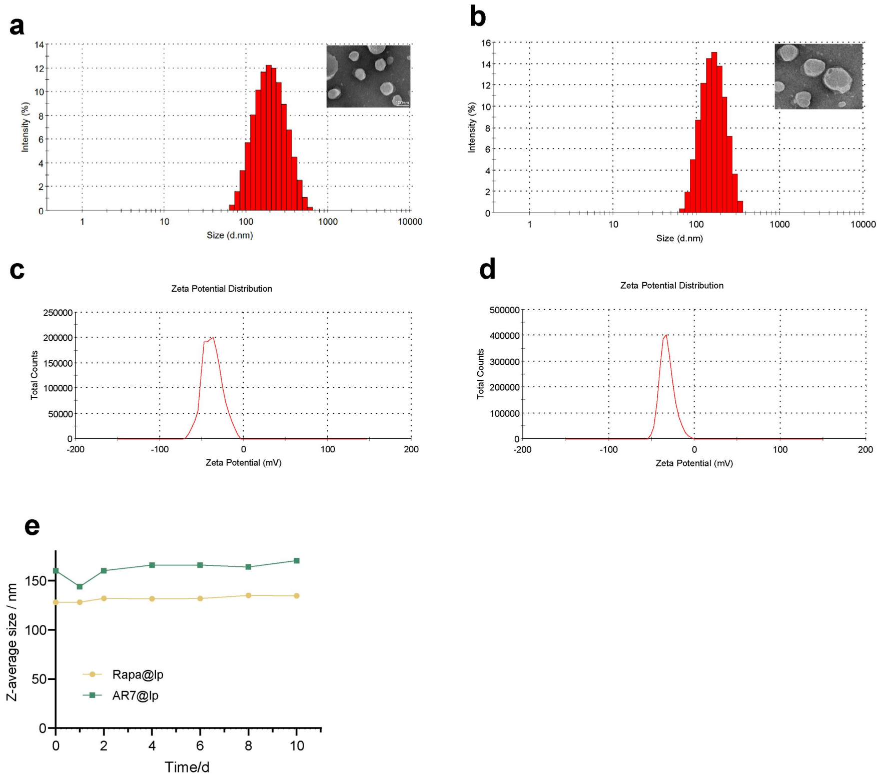

Figure. S1. Preparation and characterization of AR7@LP and Rapa@LP. **(a-b)** Particle size distribution images, TEM images of AR7@LP (a) and Rapa@LP (b). **(c-d)** Zeta potential analysis of AR7@LP (c), Rapa@LP (d). **(e)** The stability profiles for AR7@LP and Rapa@LP.

Supplementary Figure 2

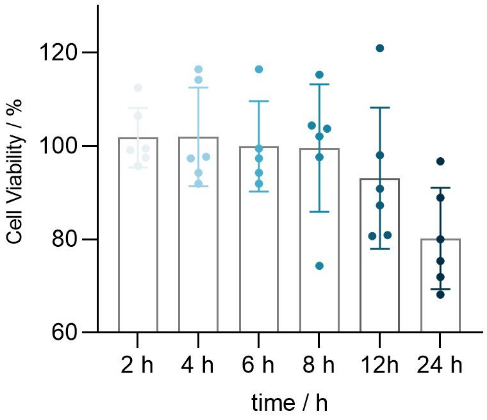

Figure. S2. Microglial viability at different internalization time points. Data are presented as mean ± SD, n=5.

Supplementary Figure 3

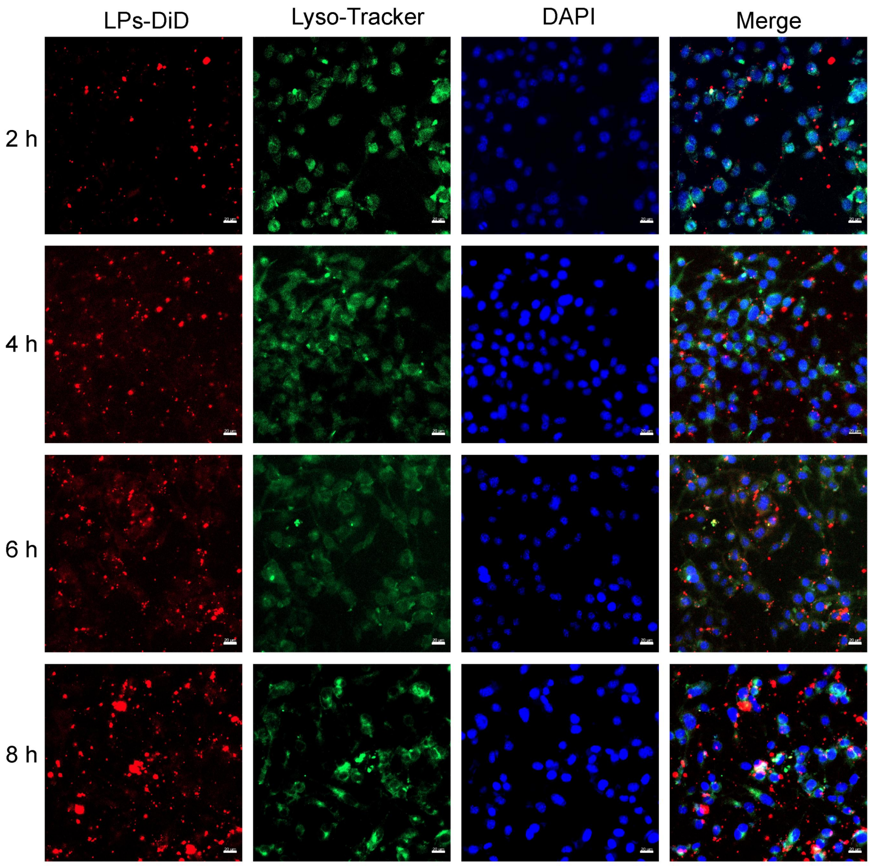

Figure. S3. Representative co-localization images of liposomes and lysosomes in microglia at different time points. Scale bars = 20 μm.

Supplementary Figure 4

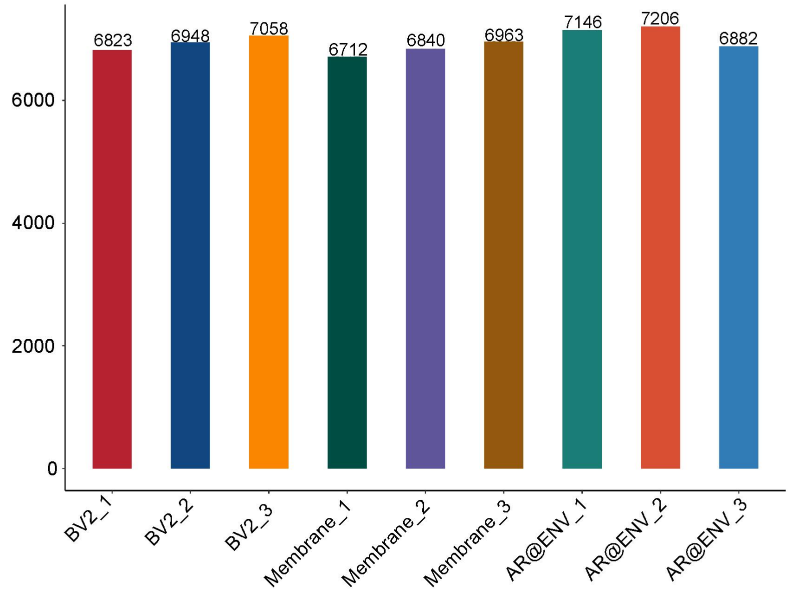

Figure. S4. Overview of Protein Identification in Samples.

Supplementary Figure 5

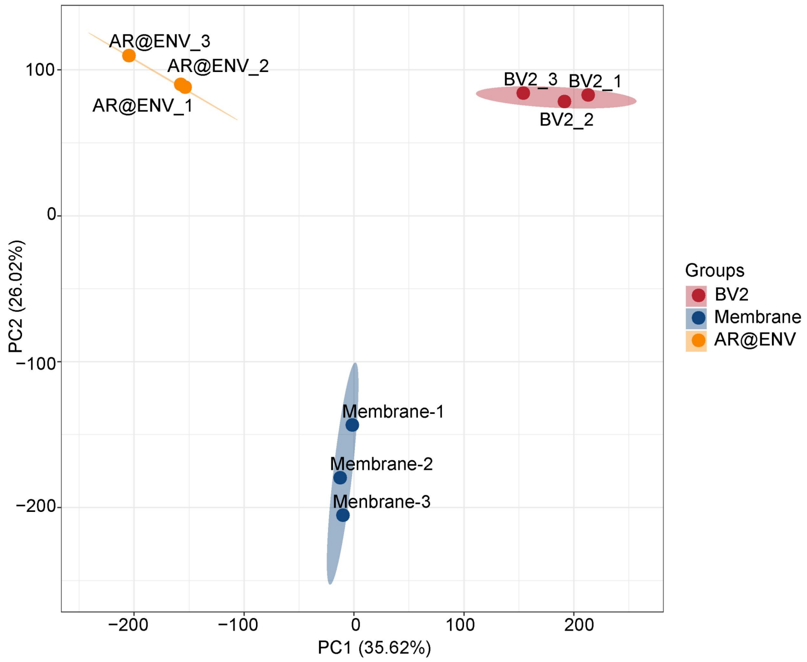

Figure. S5. Principal component analysis (PCA) of samples.

Supplementary Figure 6

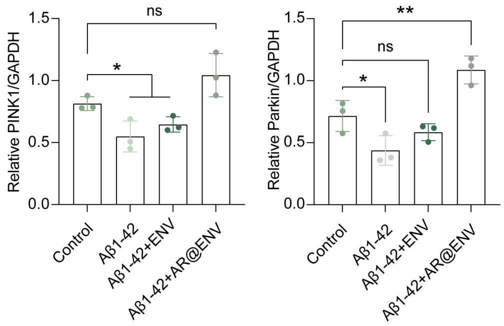

Figure. S6. Semi-quantitative analysis of the results presented in Fig 3i. presented as mean ± SD, n = 3.

Supplementary Figure 7

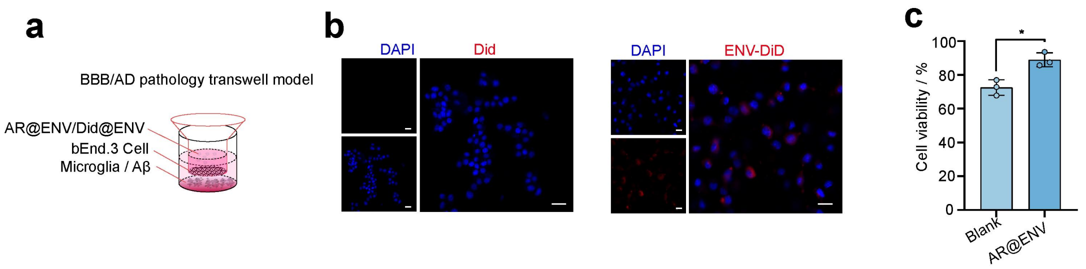

Figure. S7. (a) Schematic diagram of in vitro BBB/AD model Transwell experiment. **(b)** Representative confocal images of ENV uptake by BV2 cells in the lower chamber after crossing the BBB; Scale bar = 20 μm. **(c)** Protective effect of AR@ENV against Aβ1-42 oligomer-induced cytotoxicity in BV2 cells in the lower chamber after crossing the BBB model, presented as mean ± SD, n = 3.

Supplementary Figure 8

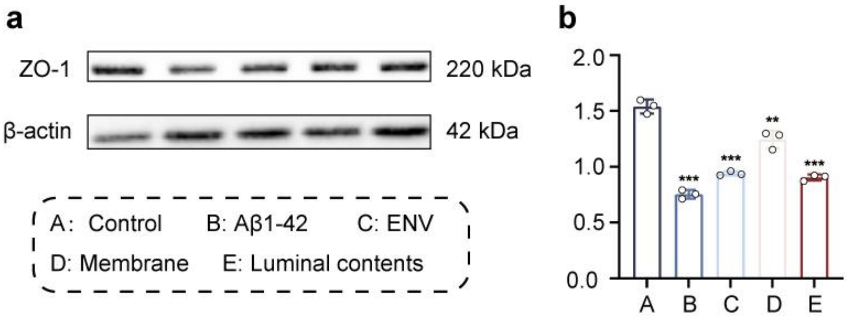

Figure. S8. Western blot analysis of ZO-1 expression. **(a)** Representative western blot images. **(b)** Semi-quantitative analysis of ZO-1/β-actin. compared with ENV group, n = 3.

Supplementary Figure 9

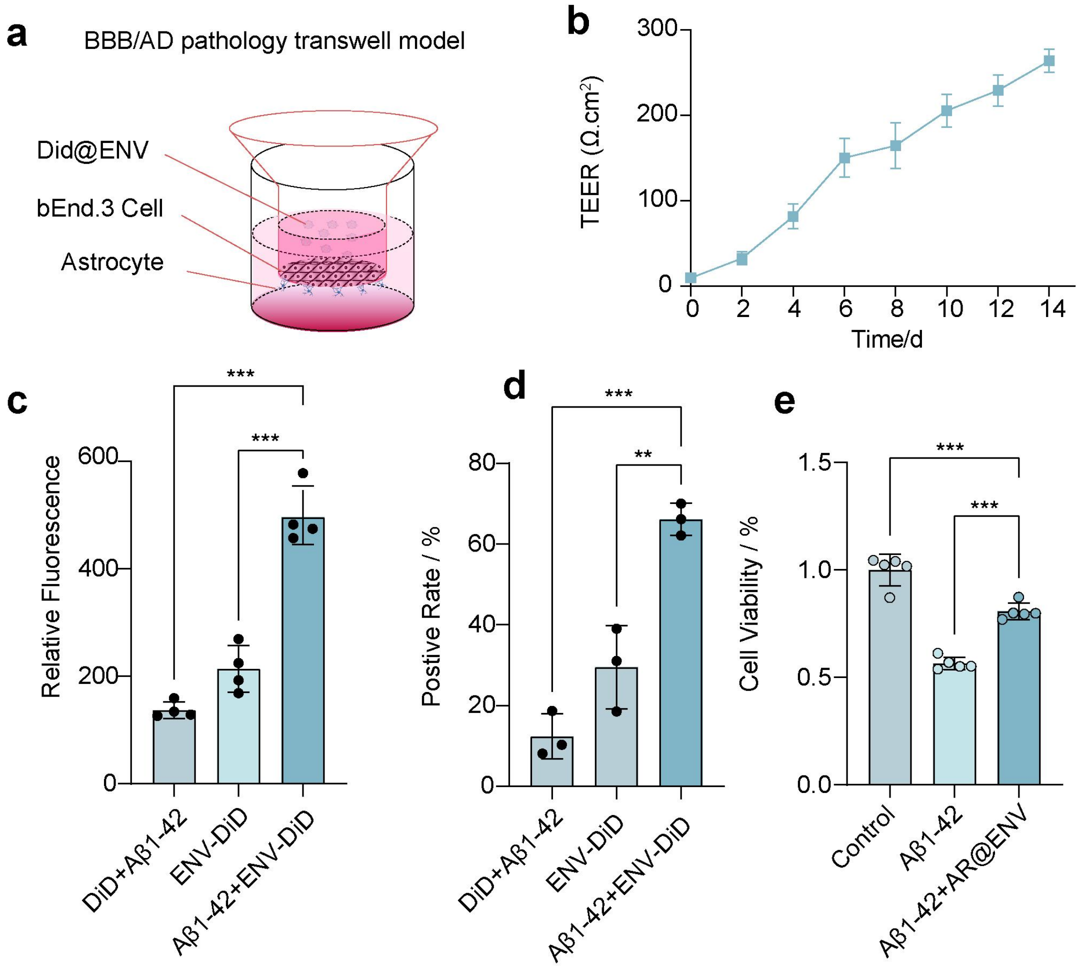

Figure. S9. *In vitro* simulation of AR@ENV crossing the blood-brain barrier (BBB) behavior. **(a)** Scheme of the in vitro BBB or Aβ1-42 induced AD BBB model using a transwell assay. (**b)** Transwell model TEER value change curve. Data are presented as mean ± SD (n=3). (**c)** Fluorescence intensity measured in the lower chamber of transwell model after different treatments. (**d)** Flow cytometry analysis of ENV uptake in neuronal cells. Data are presented as mean ± SD, n=3. (**e**) Cytoprotective effect of AR@ENV against Aβ1-42 oligomer-induced toxicity in neuronal cells following transwell traversal.

Supplementary Figure 10

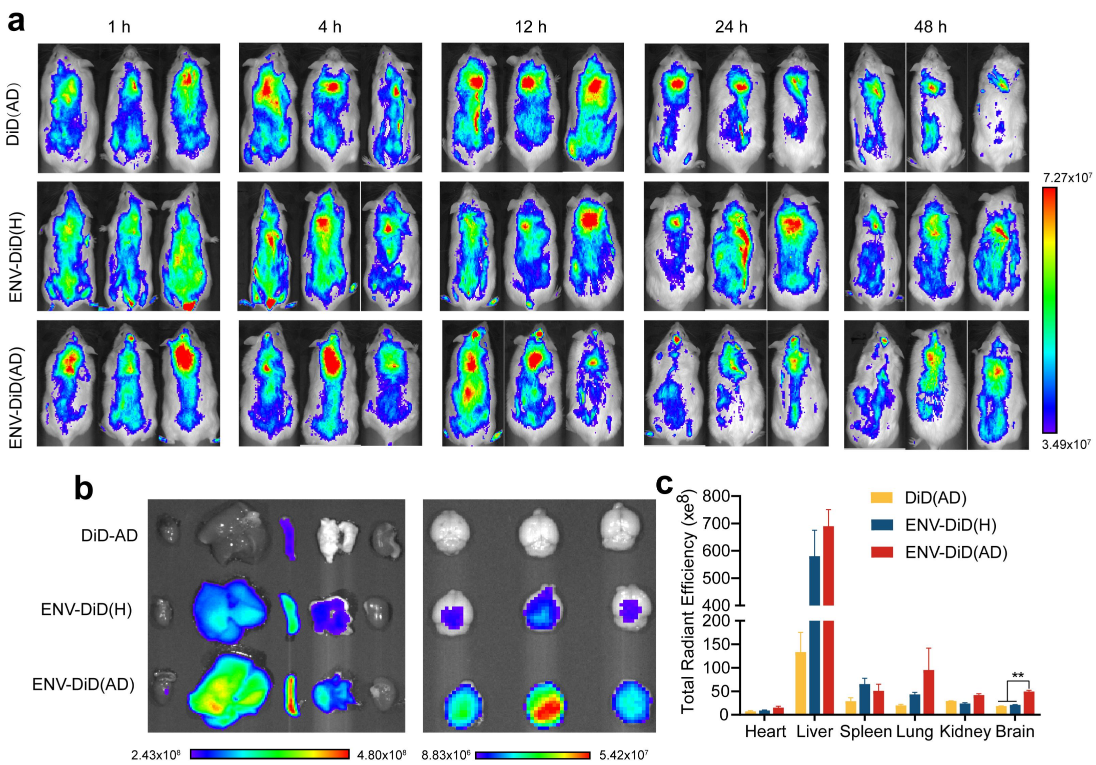

Figure. S10. In vivo distribution of ENV in Aβ1-42 injected AD mice. **(a)** In vivo imaging of AD mice and healthy Kunming mice following intravenous injection of DiD or ENV-DiD at 1, 2, 4, 12, and 24 hours. **(b)** Ex vivo imaging of major organs (left) and brain (right) from AD mice or healthy Kunming mice at 48 hours post-injection, **(c)** Semi-quantitative analysis of total fluorescence intensity in major organs, presented as mean ± SD, n=3.

Supplementary Figure 11

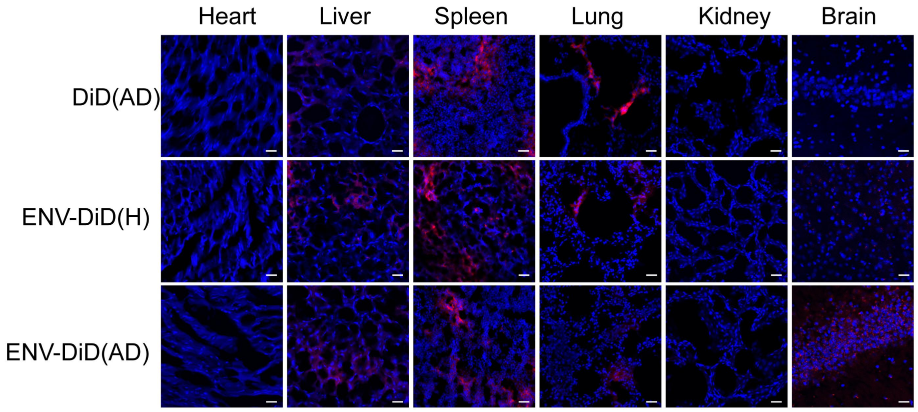

Figure. S11. Frozen tissue section images illustrating the distribution of ENV-DiD in major organs of APP/PS1 mice. Red indicates DiD-labeled ENV, with cell nuclei stained using DAPI. Scale bar: 20 μm.

Supplementary Figure 12

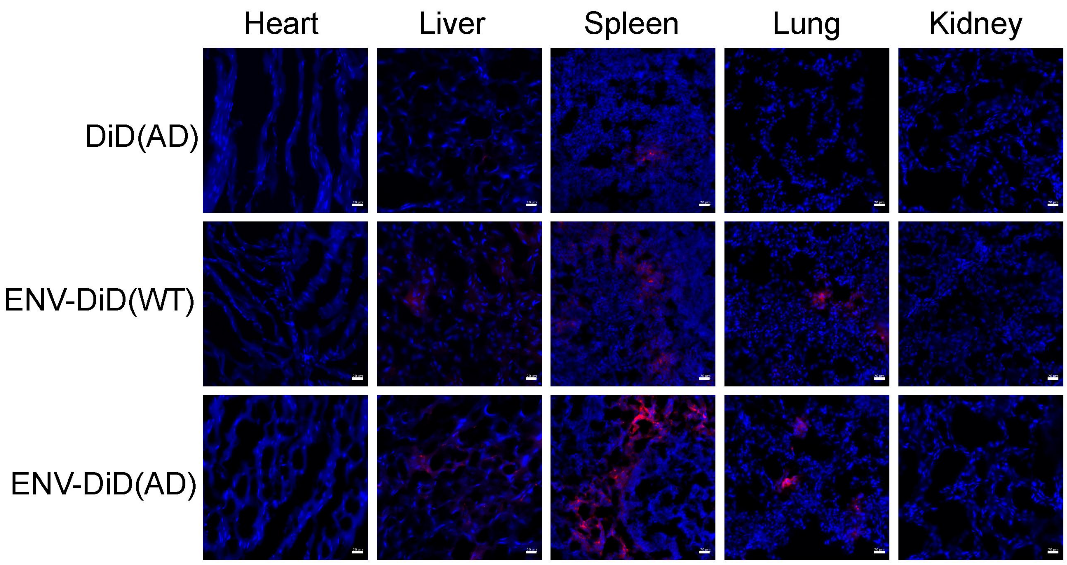

Figure. S12. Frozen tissue section images illustrating the distribution of ENV-DiD in major organs of Aβ-injected AD mice. Red indicates DiD-labeled ENV, with cell nuclei stained using DAPI. Scale bar: 20 μm.

Supplementary Figure 13

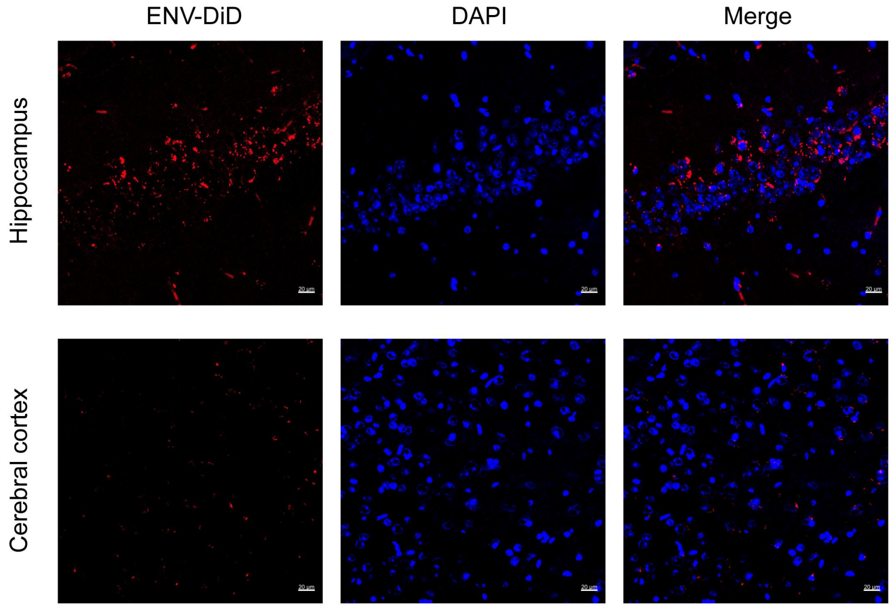

Figure. S13. Representative confocal images of ENV distribution in the hippocampal region and cerebral cortex. Scale bar: 20 μm.

Supplementary Figure 14

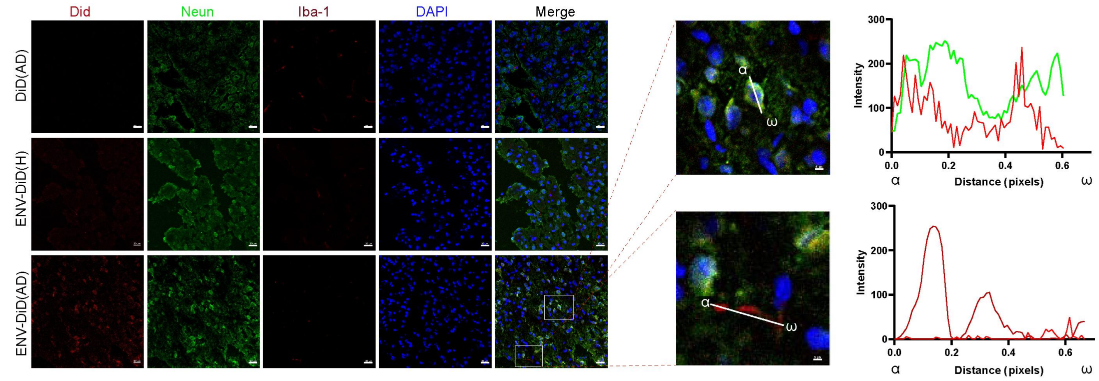

Figure. S14. Immunofluorescence images demonstrating the co-localization of ENV-DiD with neurons and microglia in the brains of Aβ-injected AD mice. Red indicates DiD-labeled ENV, green denotes neuronal cells, and purple represents microglia, with cell nuclei stained using DAPI. Representative quantitative fluorescence images of ENV-DiD co-localization with neuronal marker NeuN (top) or microglial marker Iba-1 (bottom). Scale bar: 20 μm.

Supplementary Figure 15

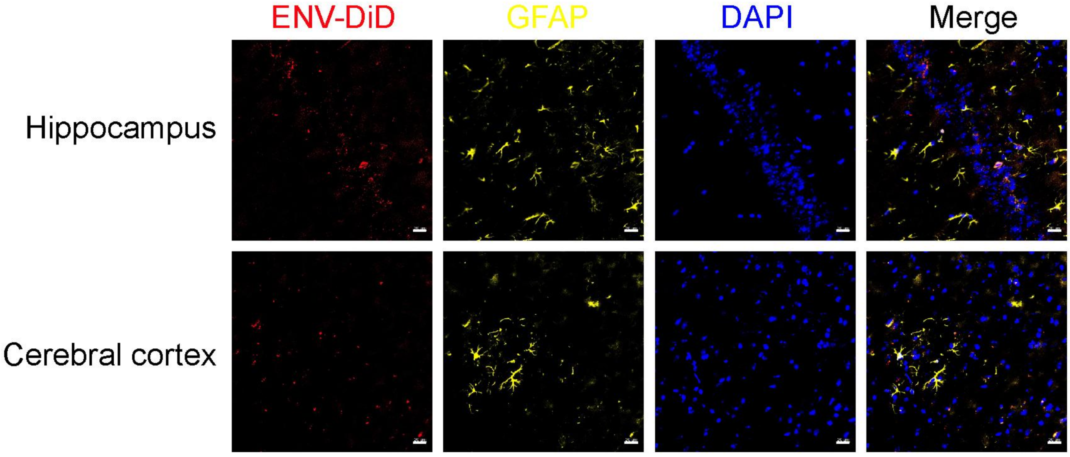

Figure. S15. Immunofluorescence images showing colocalization of ENV-DiD (red) with astrocytes (GFAP: yellow) in brain sections of APP/PS1 mice. Scale bar: 20 μm.

Supplementary Figure 16

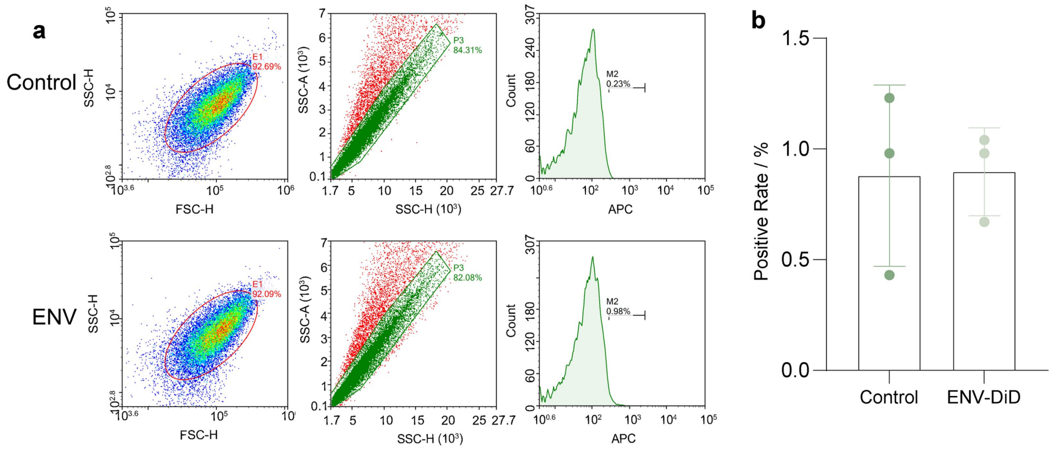

Figure. S16. (a) Representative flow cytometry images. **(b)** Semi-quantitative analysis of (a). Data are presented as mean ± SD, n=5.

Supplementary Figure 17

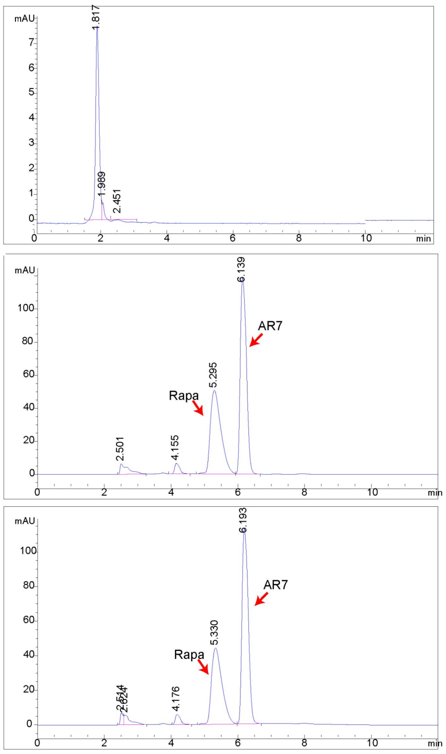

Figure. S17. Representative HPLC chromatograms of drug degradation in AR@ENV under *in vitro* simulated conditions.

Supplementary Figure 18

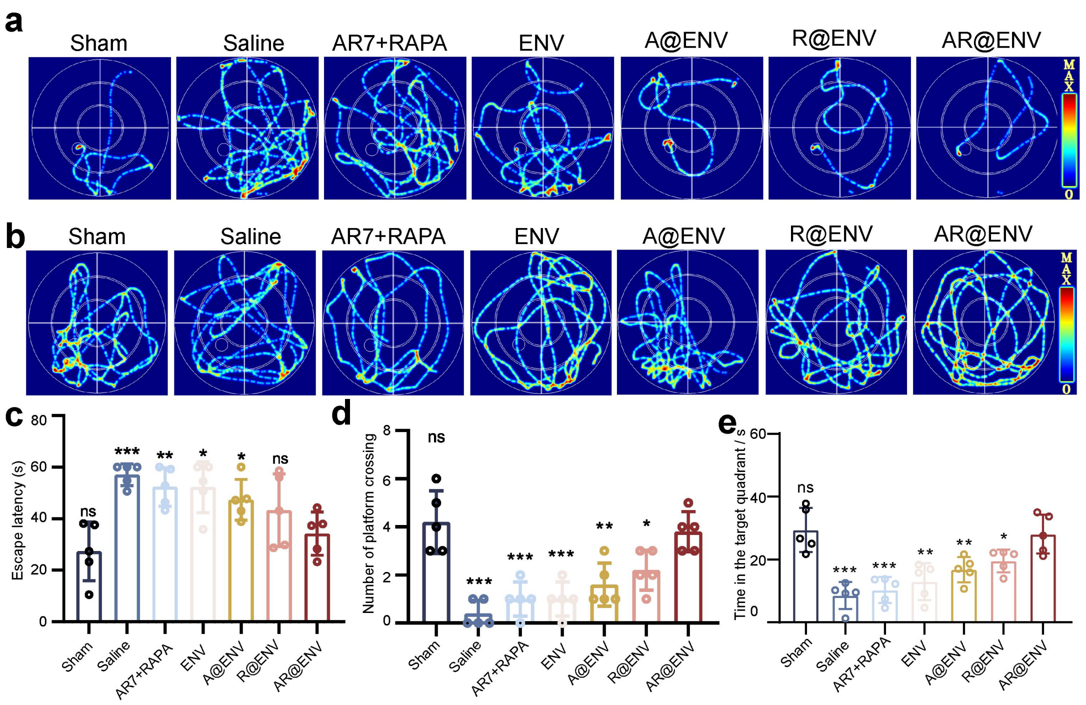

Figure. S18. Morris water maze experiment in Aβ-injected AD mice. **(a)** Representative heatmap of the spatial navigation test for mice in different treatment groups. **(b)** Representative heatmap of the spatial exploration test in Morris water maze. **(c)** Latency to enter the target platform. **(d)** Number of entries into the target platform. **(e)** Time spent in the target quadrant. All data are expressed as mean ± SD, compared with AR@ENV group, n = 5.

Supplementary Figure 19

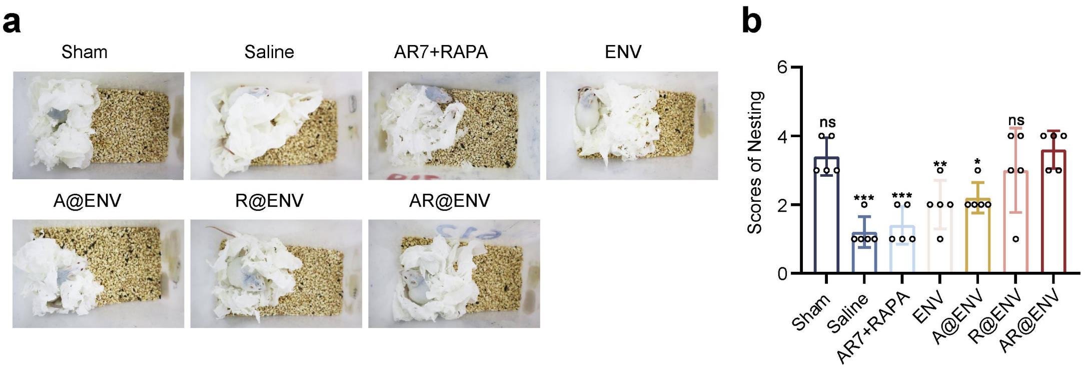

Figure. S19. Nesting-building experiment in Aβ-injected AD mice. **(a)** Representative images from the nest-building test. **(b)** Nest-building score. Data are reported as mean ± SD, compared with AR@ENV group, n = 6.

Supplementary Figure 20

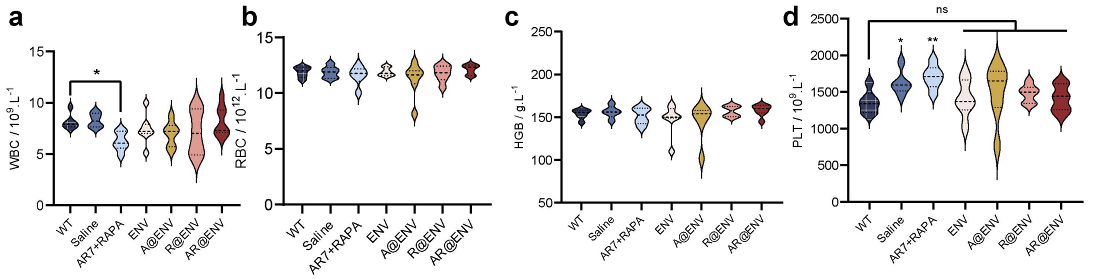

Supplementary Fig. 20. Hematological parameters of APP/PS1 mice following treatment. **(a)** WBC, **(b)** RBC, **(c)** PLT, and **(d)** HGB. Data are presented as mean ± SD, compared with AR@ENV group, n = 5.

Supplementary Figure 21

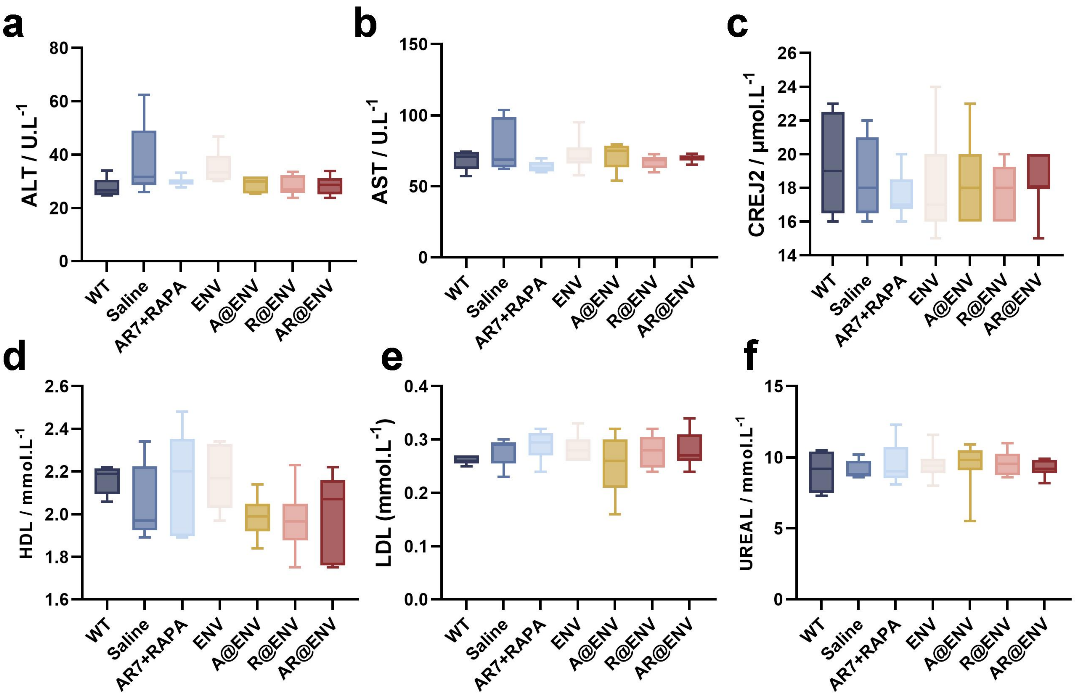

Figure. S21. Biochemical parameters across different treatment groups: **(a)** ALT, **(b)** AST, **(c)** CREJ, **(d)** HDL, **(e)** LDL and **(f)** UREAL. Data are presented as mean ± SD, compared with AR@ENV group. n = 5.

Supplementary Figure 22

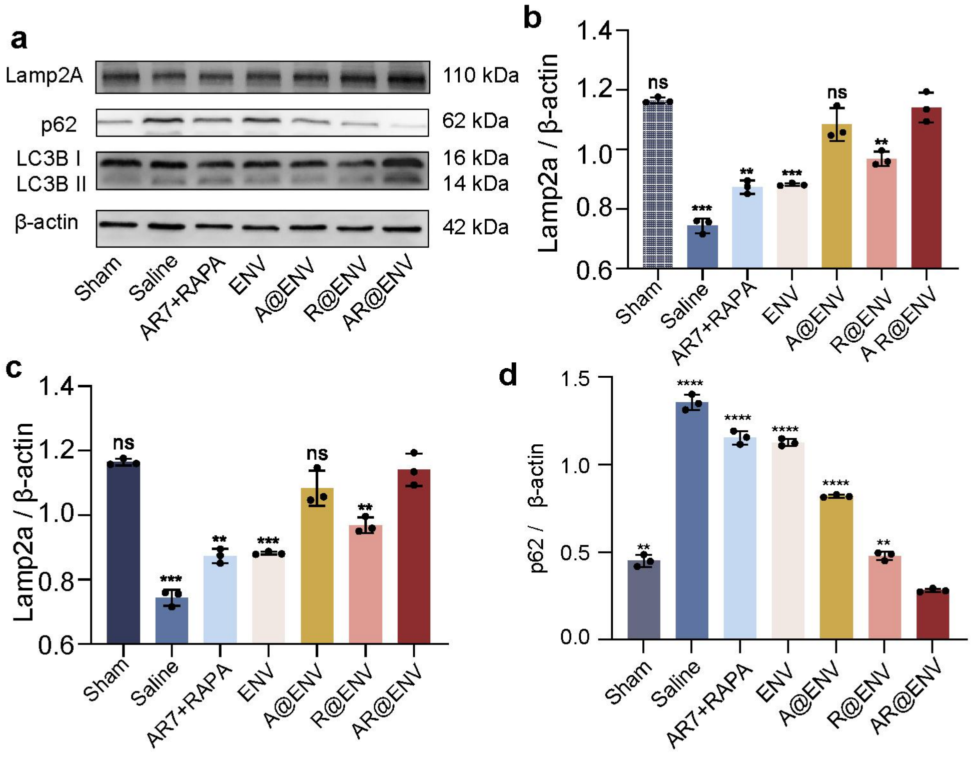

Figure. S22. Assessment of autophagy levels in brains of Aβ-injected AD mice. **(a)** Representative western blot images showing the expression of chaperone-mediated autophagy (CMA) and macroautophagy-related proteins following various treatments. **(b-d)** Semi-quantitative analysis of the CMA-related protein Lamp2A (b), macroautophagy marker p62 (c) and LC3BII/LC3BI ratio (d). Data are presented as mean ± SD, compared with AR@ENV group, n = 3.

Supplementary Figure 23

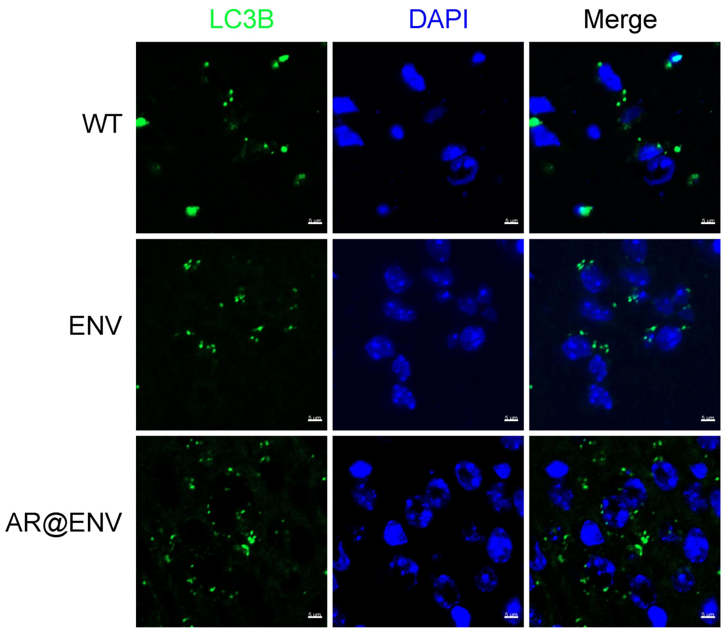

Figure. S23. Representative immunofluorescence images of autophagosomes in the hippocampal region of APP/PS1 mouse brain tissue across different treatment groups. Scale bars = 5 μm.

137 **Supplementary Figure 24**

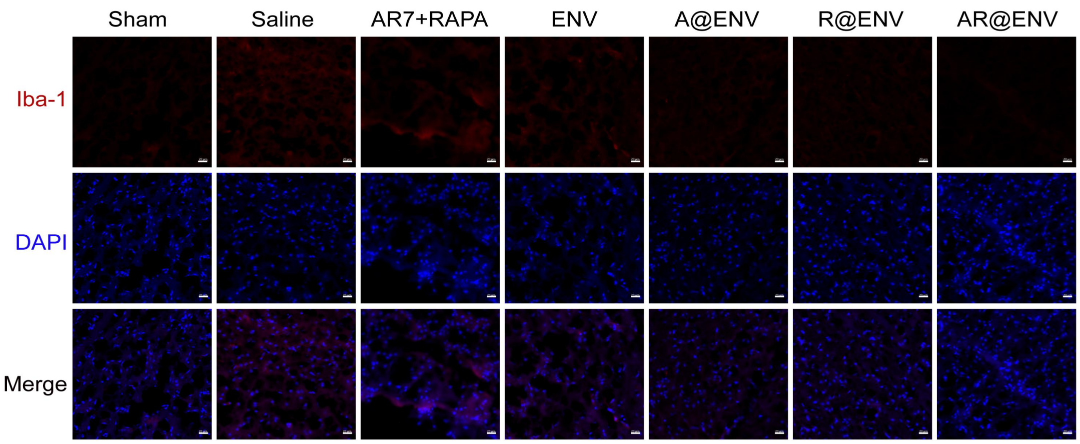

Figure. S24. Representative immunofluorescence images showed the levels of inflammatory microglia in Aβ-injected model mice after different treatments. Iba-1 protein was represented in red, and DAPI stained nuclei. Scale bar: 20 μm.

143 **Supplementary Figure 25**

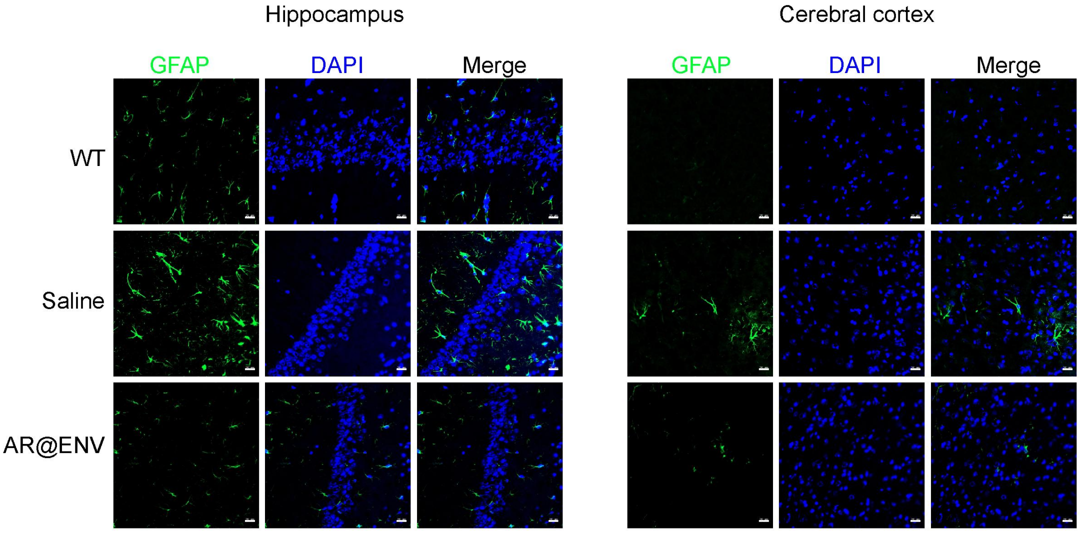

144

145 **Figure. S25.** Representative images of astrocyte proliferation in hippocampal
146 and cerebral cortex across treatment groups. Scale bar: 20 μm.

Supplementary Figure 26

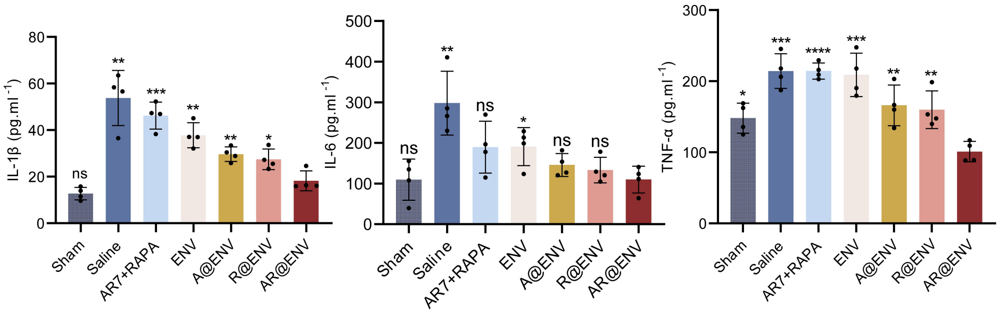

Figure. S26. Levels of inflammatory factors in brains of Aβ-injected AD mice across each treatment group were assessed using ELISA. Data were presented as mean ± SD, compared with AR@ENV group, n = 4.

Supplementary Figure 27

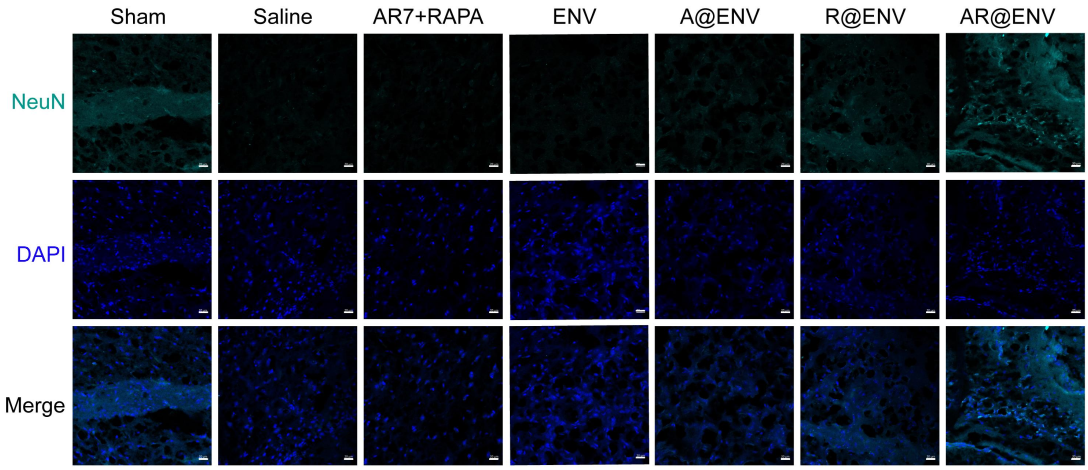

Figure. S27. Representative immunofluorescence images showed neuroprotective effects in brains of Aβ-injected AD mice from different treatment groups. NeuN was represented in blue, and DAPI stained the nuclei. Scale bar: 20 μm.

Supplementary Figure 28

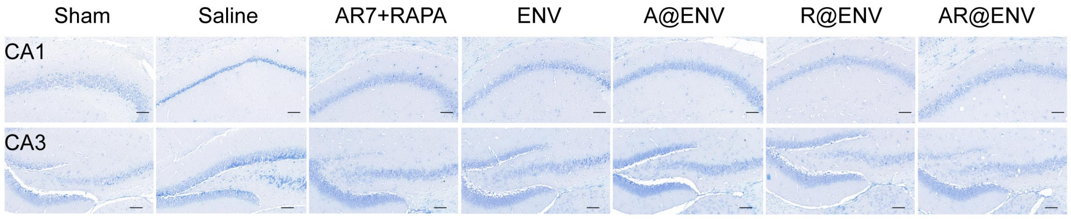

Figure. S28. Comprehensive overview of Nissl-stained of brains from Aβ-injected AD mice from different treatment groups, along with images of the hippocampal CA1 and CA3 regions. Scale bars: 50 μm.

Supplementary Figure 29

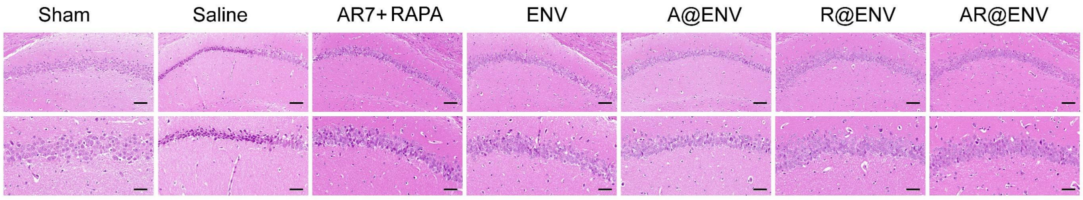

Figure. S29. Representative H&E staining images of brains from Aβ-injected AD mice across different treatment groups. Scale bar: 50 μm.

Supplementary Figure 30

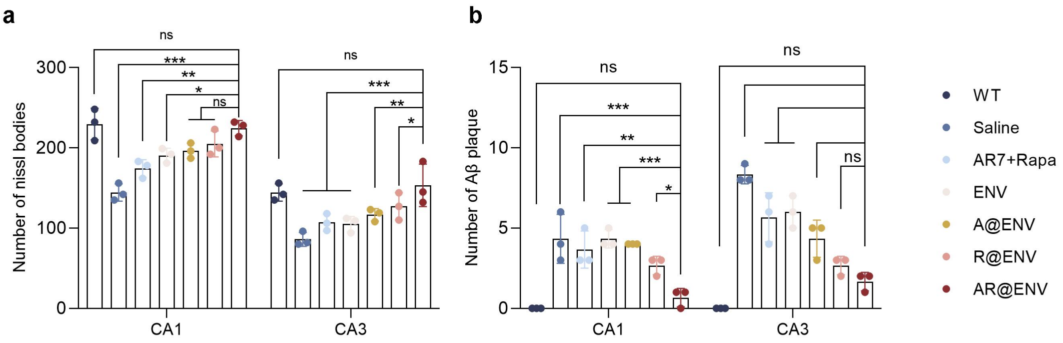

Figure. S30. Semi-quantification of Figure 7h and 7j. (**a**) Figure 7h; **(b)** Figure 7j. Data are presented as mean ± SD, n=3.

Supplementary Figure 31

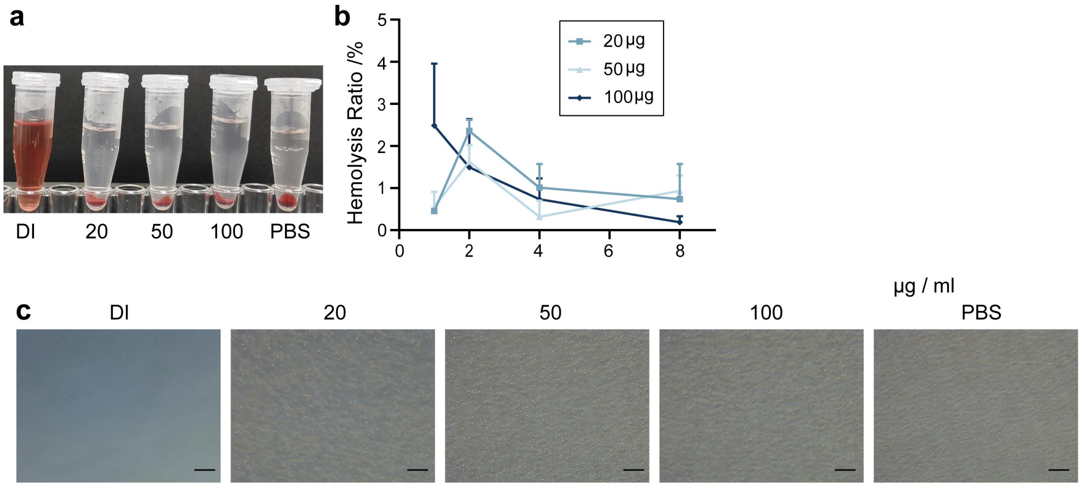

Figure. S31. Evaluation of the biocompatibility of AR@ENV. **(a)** Hemolytic rate of AR@ENV, presented as mean ± SD, n = 3. **(b)** Hemolysis ratio. **(c)** Representative images of red blood cells from different groups after 8 hours of hemolysis experiments. Scale bar = 50 μm.

Supplementary Figure 32

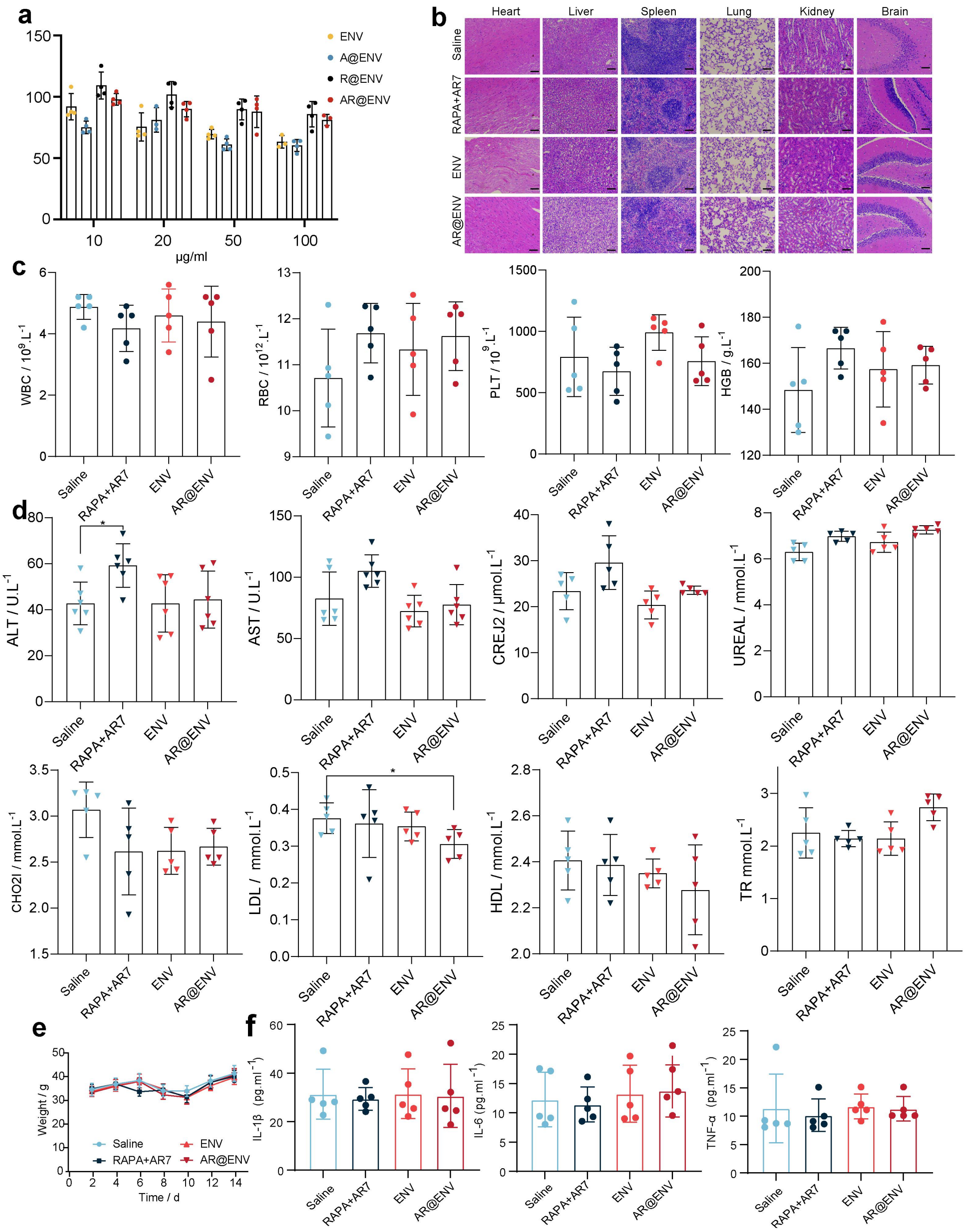

Figure. S32. Preliminary evaluation of *in vivo* safety of AR@ENV. **(a)** Cell viability assay of HT22 cells treated with different concentrations of ENV, A@ENV, R@ENV, and AR@ENV (n=4). **(b)** Representative H&E staining images of major organs from each treatment group, assessing potential histopathological alterations. Scale bar = 50 μm. **(c)** Hematological analysis of mice following long-term administration of different treatments. Data are presented as mean ± SD, n = 5. **(d)** Biochemical parameter evaluation across

different treatment groups to assess systemic toxicity. Data are presented as mean ± SD, n = 6. **(e)** Body weight fluctuations over the treatment period. Data are presented as mean ± SD, n = 5. **(f)** Serum inflammatory cytokine levels among different treatment groups. Data are presented as mean ± SD, compared with AR@ENV group, n = 5.

Supplementary Figure 33

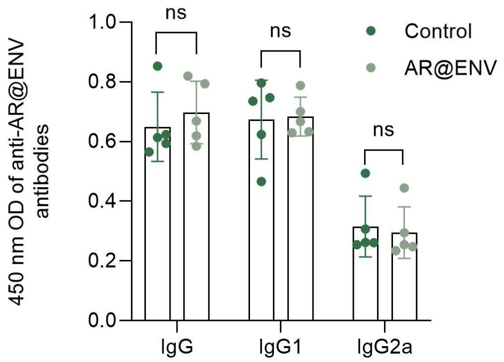

Figure. S33. Serum levels of IgG, IgG1, and IgG2a specific antibodies. Data are presented as mean ± SD, n = 5.

Supplementary Figure 34

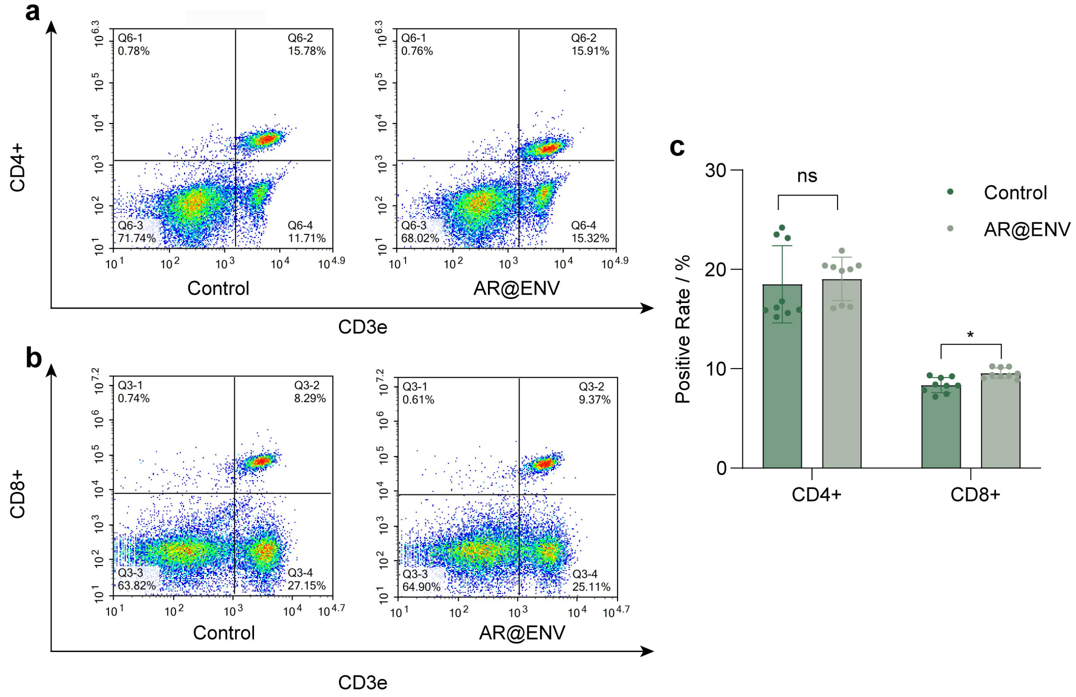

Figure. S34. Levels of CD4⁺ T cells and CD8⁺ T cells in the spleen. **(a–b)** Representative flow cytometry plots: (a) CD4⁺ T cells; (b) CD8⁺ T cells. **(c)** Quantitative analysis of panels a–b. Data are presented as mean ± SD, n = 9.

Table. S1. Size, PDI, Zeta-potential, Encapsulation Efficiency (EE)/%, and Loading efficiency (LE)/% of AR7@LP and Rapa @LP.

	Size /nm	PDI	Zeta-potential	Encapsulation Efficiency (EE)/%	Loading efficiency (LE)/%
AR7@LP	160.3 ±3.4	0.210	-37.9 ± 4.6	92.9 ± 1.3	1.3 ± 0.012
Rapa@LP	127.3 ± 2.6	0.212	-30.8 ± 1.5	84.5 ± 1.5	3.91 ± 0.058

Table. S2. Drug loading capacity of AR@ENV at different internalization time points.

Time	AR7 loading efficiency /μg	Rapa loading efficiency /μg
2 h	3.7 ± 0.25	1.1 ± 0.15
4 h	8.71 ± 0.46	2.6 ± 0.12
6 h	12.7 ± 0.21	4.12 ± 0.06
8 h	19.4 ± 0.37	6.75 ± 0.05
12 h	20.2 ± 1.2	7.31 ± 0.09

Table. S3. Protein concentration, particle concentration, and drug loading capacity of three batches of AR@ENV.

	Protein concentration ($mg.ml^{-1}$)	Particle concentration/ ($particles.ml^{-1}$)	AR7 loading efficiency /μg	Rapa loading efficiency /μg
1	0.95±0.0017	$4.8*e^{11}$	19.4 ± 0.37	6.75 ± 0.05
2	0.98±0.0097	$4.9*e^{11}$	18.7 ± 0.07	6.24 ± 0.12
3	1.08±0.032	$5.1*e^{11}$	19.9 ± 0.22	7.02 ± 0.34

Table. S4. Overview of DIA-Based Protein Identification.

Name	Peptide	Identified protein
ALL	94062	7771

Table. S5. Differential Protein Screening.

Compared samples	Num. of Total Quant.	Regulated type	fold change > 1.2	fold change > 1.3	fold change > 1.5	fold change > 2.0
AR@ENV.vs.BV2	6931	Up-regulated	1792	1773	1696	1396
		Down-regulated	2074	2064	1967	1925
AR@ENV.vs.Membrane	6836	Up-regulated	1469	1444	1343	1018
		Down-regulated	1820	1802	1681	1187

Table. S6. Quantification of rapamycin and AR7 degradation in AR@ENV by HPLC.

	Peak Area（mAU *s）						degradation percentage/%
	0 h			8 h			
Rapa	1107.1	1114.9	1108	965.9	974.1	963	12.8 ±2.4
AR7	1519.8	1525.0	1516.3	1460.3	1465.7	1459.5	3.8±0.9

Table. S7. Antibodies used for immunofluorescence staining and western blotting

Antibody	Supplier name	Catalogue number	Dilution ratio
Lamp2A	Abcam	ab240018	1： 2000
TSG101	Huabio	ET1701-59	1： 2000
CD9	Huabio	HA721533	1： 2000
P62	Abways	CY9081	1： 5000
LC3B	Abways	CY5992	1： 2000
Iba-1	Abcam	ab178846	1： 500
Neun	Abcam	Ab177487	1： 500
GAPDH	Abways	AB0037	1:10000
β-actin	Affinity	T0022	1:10000
ZO-1	Abcam	ab307799	1:500
PINK1	Huabio	ER1706-27	1:1000
Parkin	Huabio	ET1702-60	1:1000
CX3CR1	Abcam	ab308613	1:1000
IgG	Abclonal	AB0102	1:10000
IgG1	Abclonal	AS066	1:2000
IgG2a	Abclonal	AS065	1:2000

Table. S8. Primers used for qRT-PCR

Gene	Primer	Sequence
Mtor	F	TGGCATAACAGATCCTGACCC (21)
Mtor	R	CAGGGATGCCAAGACACAGT (20)
AKT1	F	CCGCCTGATCAAGTTCTCCTA (21)
AKT1	R	CAGCGCATCCGACAAACAAA (20)
GAPDH	F	CATGAGAAGTATGACAACAG (20)
GAPDH	R	ATGAGTCCTTCCACGATA (18)
Pten	F	CAAGAGGATGGATTCGACTTAGAC (24)
Pten	R	AAAGGATACTGTGCAACTCTGC (22)
