# Supplementary figures and images for: Microglia-derived nanovesicles synchronize macroautophagy and chaperone-mediated autophagy for Alzheimer’s disease therapy
Source: Signal Transduct Target Ther. 2025 Nov 3;10:360. doi: 10.1038/s41392-025-02453-y (PMC12583606; doi:10.1038/s41392-025-02453-y)

Figure 2a

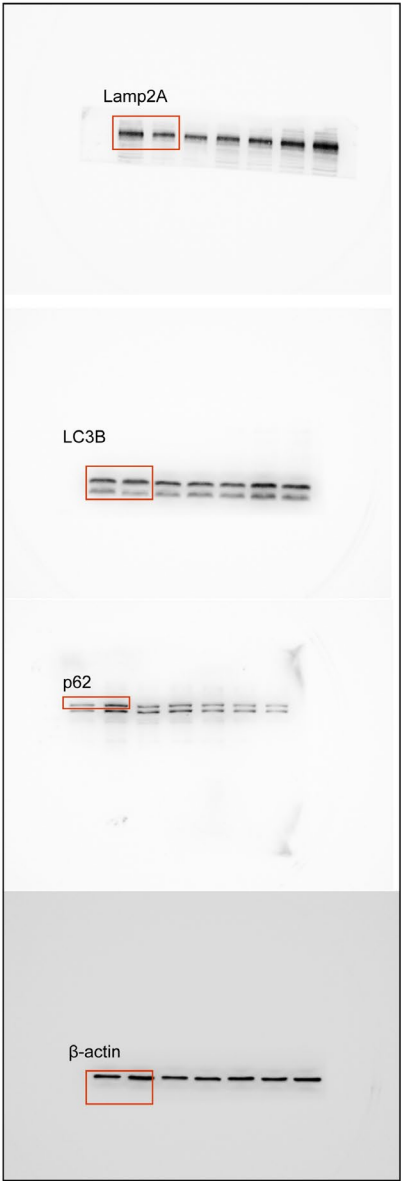

Figure 2c

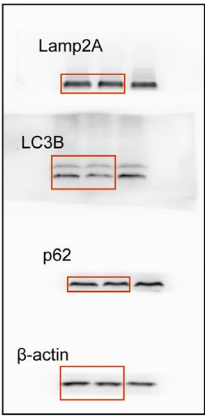

Figure 2j

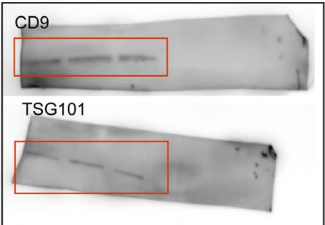

Figure 2l

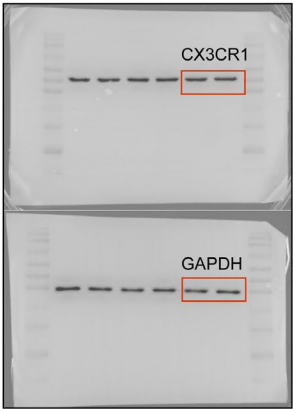

Figure 3b

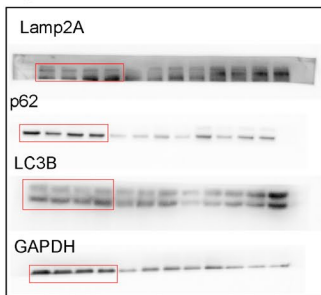

Figure 3i

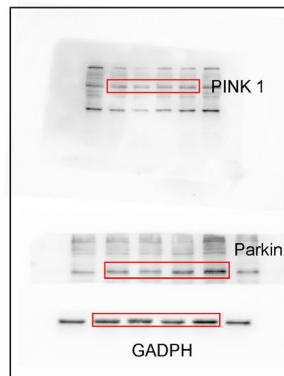

Figure 7a

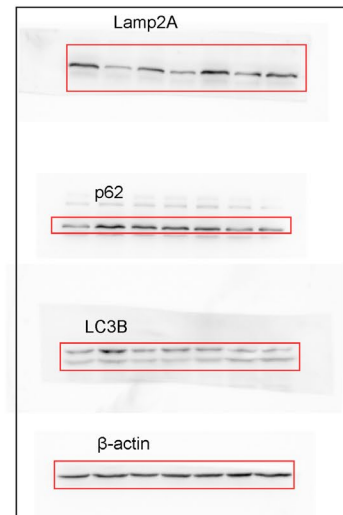

Figure S8a

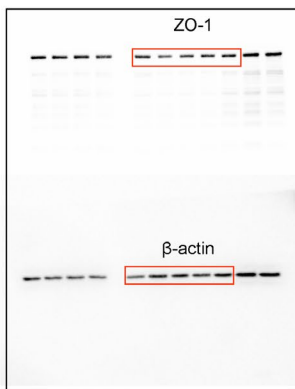

Figure S22a

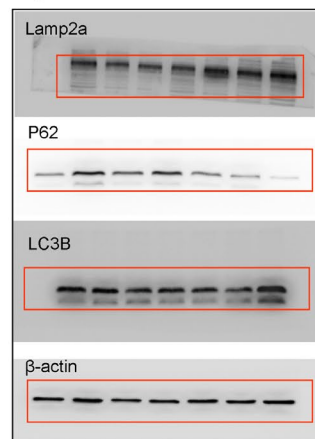

Supplement: Supplementary file 2 — Uncropped blot [file 41392_2025_2453_MOESM2_ESM.pdf]
